# Effects of serum proteins on corrosion rates and product bioabsorbability of biodegradable metals

**DOI:** 10.1093/rb/rbad112

**Published:** 2023-12-12

**Authors:** Hongjie Zhang, Xin Li, Zehua Qu, Wanqian Zhang, Qunsong Wang, Dinglingge Cao, Yaoben Wang, Xin Wang, Yang Wang, Lin Yu, Jiandong Ding

**Affiliations:** State Key Laboratory of Molecular Engineering of Polymers, Department of Macromolecular Science, Fudan University, Shanghai 200438, China; State Key Laboratory of Molecular Engineering of Polymers, Department of Macromolecular Science, Fudan University, Shanghai 200438, China; State Key Laboratory of Molecular Engineering of Polymers, Department of Macromolecular Science, Fudan University, Shanghai 200438, China; State Key Laboratory of Molecular Engineering of Polymers, Department of Macromolecular Science, Fudan University, Shanghai 200438, China; State Key Laboratory of Molecular Engineering of Polymers, Department of Macromolecular Science, Fudan University, Shanghai 200438, China; State Key Laboratory of Molecular Engineering of Polymers, Department of Macromolecular Science, Fudan University, Shanghai 200438, China; State Key Laboratory of Molecular Engineering of Polymers, Department of Macromolecular Science, Fudan University, Shanghai 200438, China; State Key Laboratory of Molecular Engineering of Polymers, Department of Macromolecular Science, Fudan University, Shanghai 200438, China; State Key Laboratory of Molecular Engineering of Polymers, Department of Macromolecular Science, Fudan University, Shanghai 200438, China; State Key Laboratory of Molecular Engineering of Polymers, Department of Macromolecular Science, Fudan University, Shanghai 200438, China; State Key Laboratory of Molecular Engineering of Polymers, Department of Macromolecular Science, Fudan University, Shanghai 200438, China

**Keywords:** biodegradable metal, macromolecule, albumin, corrosion, bioabsorbability, iron, polylactide, magnesium, zinc

## Abstract

Corrodible metals are the newest kind of biodegradable materials and raise a new problem of the corrosion products. However, the removal of the precipitated products has been unclear and even largely ignored in publications. Herein, we find that albumin, an abundant macromolecule in serum, enhances the solubility of corrosion products of iron in blood mimetic Hank’s solution significantly. This is universal for other main biodegradable metals such as magnesium, zinc and polyester-coated iron. Albumin also influences corrosion rates in diverse trends in Hank’s solution and normal saline. Based on quantitative study theoretically and experimentally, both the effects on corrosion rates and soluble fractions are interpreted by a unified mechanism, and the key factor leading to different corrosion behaviors in corrosion media is the interference of albumin to the Ca/P passivation layer on the metal surface. This work has illustrated that the interactions between metals and media macromolecules should be taken into consideration in the design of the next-generation metal-based biodegradable medical devices in the formulism of precision medicine. The improved Hank’s solution in the presence of albumin and with a higher content of initial calcium salt is suggested to access biodegradable metals potentially for cardiovascular medical devices, where the content of calcium salt is calculated after consideration of chelating of calcium ions by albumin, resulting in the physiological concentration of free calcium ions.

## Graphical Abstract

**Figure:**
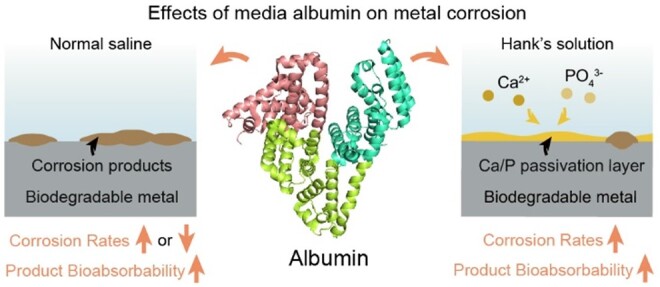

## Introduction

The new-generation biomaterials are expected to be biodegradable [1]. While a polymer degrades via chain scission and a bioceramic degrades via dissolution as usual, the biodegradation of a metal takes place via corrosion. Anticorrosion has, for at least one century, been a hard work in many application fields [2, 3], but such a situation is changing because the development of the metal-based biodegradable medical devices relies on metal corrosion in physiological aqueous media. The corresponding main chemical reactions are shown in Supplementary Figure S1. The complete biodegradation with no implant residues would assist tissue regeneration. Different from biodegradable polymers and non-metallic inorganics, biodegradable metals have a particular concern, namely, how to remove the corrosion products at precipitate state. While it is much desired to alleviate insoluble corrosion products, the corresponding fundamental study or feasible approach under biomimicking media is rather limited so far.

With the minimal toxic response and mechanical properties to match the target tissue, the biodegradable metals such as magnesium, iron, zinc and some alloys have potentially wide medical applications, particularly in cardiovascular and musculoskeletal implants [4–10]. The magnesium-based biodegradable coronary stents, Magmaris (Biotronik AG, Bulach, Switzerland), the iron-based biodegradable stents and metal-polymer composite stents have recently exhibited their safety and performance in preclinical and clinical studies [11–16]. In order to promote large scale clinical applications of the newest-generation biodegradable material, it is urgent to accurately predict the degradation rate of a corrodible metal and reveal the cues to influence the amount of insoluble corrosion products in biomimetic aqueous media.

The degradation of a metal is highly influenced by the environmental cues including physical, chemical and biological ones [17–22]. It has known that small molecules and ions can influence metal corrosion [23–28]. As early as in 1982, Clark and Williams addressed the question of the effects of proteins on metallic corrosion and pioneered the study of unwelcome corrosion at that time for those permanent metals including aluminum, chromium, cobalt, copper, molybdenum, nickel, titanium and Co–Cr–Mo alloy [29]. Nevertheless, valuable yet only a little effort has been made to investigate metal corrosion in the media containing biomacromolecules since then [30–35], possibly because biodegradable metals representative by iron, magnesium and zinc have been emerged as a critical topic only recently. Both increased and decreased corrosion rates have been reported even with the same protein [36, 37], and thus it is much required to afford a unified interpretation. More importantly, previous relevant study focuses on the effects of proteins on corrosion rates, but ignores the effects on corrosion products, especially on the bioabsorbability of corrosion products, which is essential for medical applications of a biodegradable metal.

**Figure 1.**
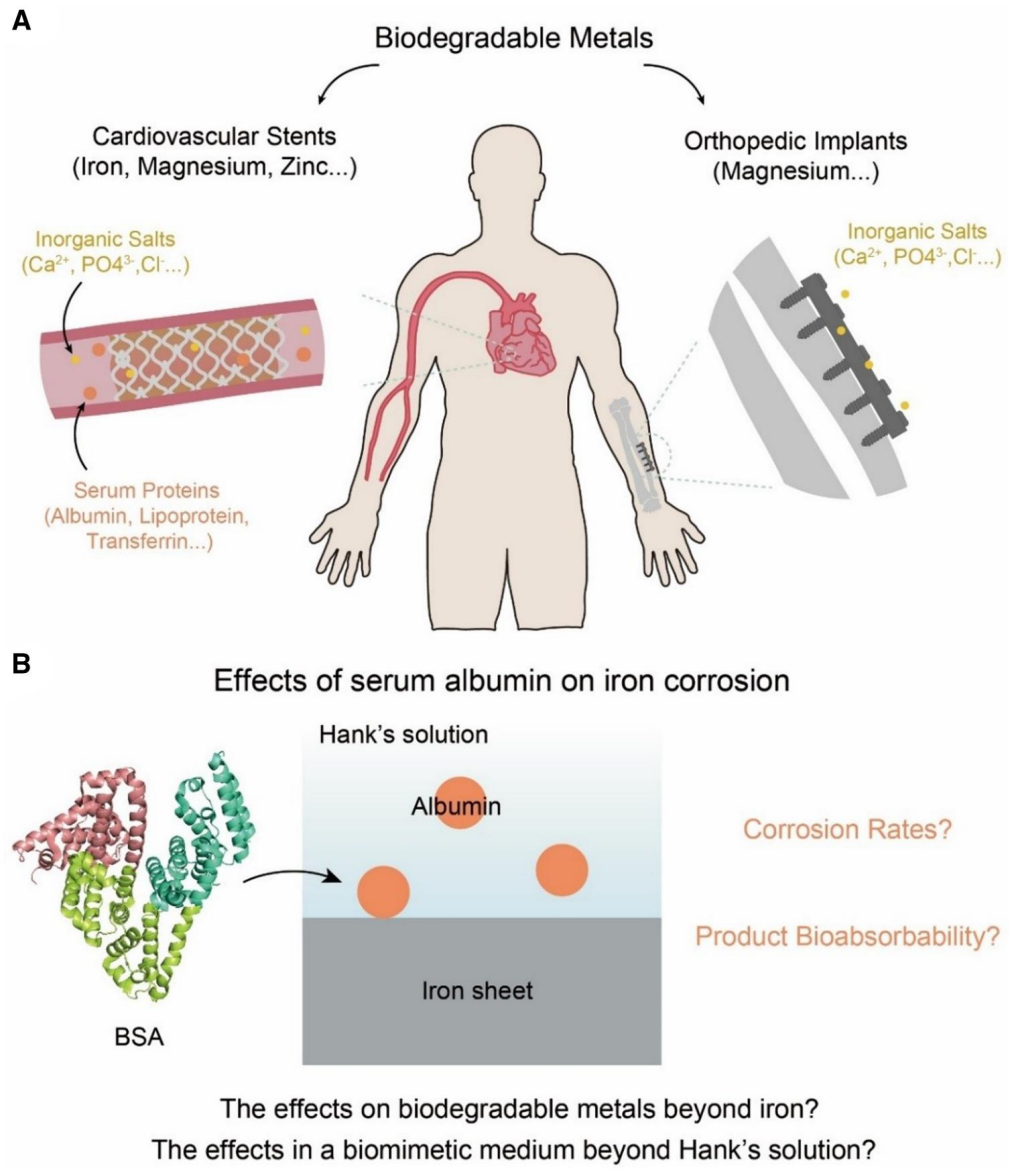
Schematic diagram of the fundamental study of the effects of serum proteins on metal corrosion. (**A**) Illustration of inorganic salts and serum proteins to influence the degradation behaviors of biodegradable metals widely used in cardiovascular stents and other medical devices. (**B**) Diagram of bovine serum albumin (BSA) and the main scientific questions in the present study.

Herein, the effects of media macromolecules on corrosion rates and corrosion products were comprehensively examined via quantitative study theoretically and experimentally. As shown in Figure 1, the cardiovascular environment was chosen as the object of our study with Hank’s solution as the pseudo-physiological solution. Albumin, the most abundant protein in serum, was chosen as the representative of the relevant biomacromolecules. Iron, a biodegradable metal with excellent mechanical properties, was chosen as the main metal type. Our experiments illustrated that albumin influenced metal corrosion through its interaction with both the solution components and the metal surface. The albumin was found to improve the bioabsorbability of corrosion products for the first time, which was accounted for from quantitative investigation of the complexation of metal cations to serum proteins.

## Materials and methods

### Sample preparation and *in vitro* immersion experiments

Iron sheets of 200 μm (iron content > 99.99%), magnesium sheets of 300 μm (magnesium content > 99.99%) and zinc sheets of 300 μm (zinc content > 99.99%) were cut into 10 mm × 10 mm. Parts of the iron sheets were welded onto copper wires and sealed by epoxy resin to prepare iron electrodes. Then, the embedded electrodes and the above metal sheets were polished by SiC paper of 800, 1500, 2000 and 3000 grits. After that, these samples were ultrasonically cleaned in acetone and alcohol and then quickly dried by nitrogen gas.

A polylactide (PLA) solution of 0.01 g/ml was prepared by dissolving poly(d,l-lactic acid) with molecular weight (MW) of 60 kDa (Jinan Daigang Biomaterial Co., Ltd, China) into ethyl acetate. The polymeric solution was ultrasonically sprayed on the cleaned iron sheets to prepare a PLA coating with an ultrasonic spray integration system (Shanghai Ruidu Photoelectric Co., Ltd, China), and then the PLA-coated iron sheets were placed in the fume cupboard overnight to evaporate solvent. The spraying cycle proceeded 10 times, and the dispense rate of the syringe pump was 0.05 ml/min. The peripheral edges and back sides of the above bare metal sheets and PLA-coated iron sheets were sealed by silicone to leave only one side exposed.

The chemical compositions of the main test solutions are listed in Supplementary Table S1. Each sheet was immersed in 10 ml test solution, and each electrode was immersed in 100 ml test solution in a water bath shaker (37°C, 50 rpm). The pH was adjusted to 7.35–7.45 before the immersion experiments.

### Characterization of BSA

The structure data of bovine serum albumin (BSA) and human serum albumin (HSA) are from the protein data bank [38, 39]. The absolute MW of the BSA (Sigma-Aldrich) was measured with the matrix-assisted laser desorption ionization time-of-flight (MALDI-TOF) mass spectrometer (5800, AB SCIEX, USA). The sample was mixed with sinapic acid. The mixture was deposited on the MALDI target. The positive-ion mass spectrum was recorded in the linear mode. The contents of iron ion, magnesium ion and zinc ion impurities in BSA were determined with inductively coupled plasma atomic emission spectrometry (ICP-AES, iCAP 7400, Thermo Fisher, USA) after the BSA was dissolved in Milli-Q water.

### Scanning electron microscopy observation

After being immersed for 24 h, the iron sheets were rinsed well in Milli-Q water and vacuum-dried, and then the surface morphologies were photographed with a digital camera and an electron microscope (LaB6-SEM, VEGA 3 XMU, TESCAN, Germany). The surface atomic composition was semi-quantified by an energy-dispersive spectrometer (EDS) instrument. The iron sheets were then ultrasonically cleaned in tartaric acid (4 wt.%) for 20 s to remove corrosion products. The samples were all gold-sputtered prior to scanning electron microscopy (SEM) observation. The corrosion coverage was quantified by the corrosion pit area and the exposed iron sheet area acquired by the SEM images without corrosion product.

### Determination of corrosion mass and corrosion rate

After *in vitro* immersion experiments, the supernatant was collected by centrifuging the test solution (3000 rpm, 5 min). The calculation of the corrosion mass of soluble products was based on the exposed area of the metal sheet, the volume of the test solution and the metal ion concentration of the supernatant measured by ICP-AES. The metal sheets were immersed in the appropriate chemical cleaning solution. The clean solution was then mixed with the precipitates to determine the total mass of insoluble products by ICP-AES. The cleaning solution for the iron sheets was tartaric acid (4 wt.%), that for magnesium was 150 g CrO_3_ + 10 g Ag_2_CrO_4_ + 1000 ml Milli-Q water, and that for zinc alternated 150 ml NH_4_OH + 1000 ml Milli-Q water with 50 g CrO_3_ + 10 g AgNO_3_ + 1000 ml Milli-Q water according to the ASTM G1-03. The corrosion rates of iron sheets were calculated by the corrosion mass at 24 h. According to the ASTM G1-03, standard practice for preparing, cleaning and evaluating corrosion test specimens, corrosion rate in unit of mm/year is equal to 87* *600 × *m*/(*t *×* d*), where *m* denotes corrosion mass under a given project area (g/cm^2^), *t* denotes duration (h) of the corrosion test, and *d* is the density (g/cm^3^) of the sample.

### Raman spectroscopy and dynamic light scattering to characterize the corrosion products

Raman spectroscopy was applied to characterize the insoluble corrosion products on iron sheets immersed in Hank’s solution with and without BSA for 24 h. The measurements were carried out three times, 60 s each time with a Raman spectrometer (XploRA, HORIBA JobinYvon, France) with a 532-nm laser, a 100× lens and a 600 g/mm grating. LabSpec software was used to process and analyze the spectra.

A dynamic light scattering (DLS) spectrometer (Zetasizer Nano ZS90, Malvern, UK) was applied to characterize the particle size distribution of the soluble corrosion products of iron sheets immersed in Hank’s solution with BSA for 24 h. Hank’s solution with 40.0 g/L BSA yet without immersing of the iron sheets was selected as the control. The light source was a 532-nm argon ion laser and the scattering angle was 90°. The measurements were carried out 20 times at 25°C. Prior to the measurements, the solutions were filtered by 0.22-μm filters.

### Equilibrium dialysis method to calculate the apparent association constant of the soluble Fe–BSA coordination complex

The metal indicators, 1,10-phenanthroline for ferrous ion and sulfosalicylic acid for ferric ion, were applied to determine the valence state of iron in the soluble iron corrosion products, abbreviate as Fe–BSA. Hank’s solution with BSA after immersing the iron sheet was filtered by a 0.22-μm filter, then mixed with 1,10-phenanthroline aqueous solution or sulfosalicylic acid aqueous solution. After that, these mixed solutions were adjusted to pH 2–9 or pH 9–11. As a control, ferrous sulfate solution (250 μM) and ferric chloride (250 μM) went through the same operations as above. These four solutions were tested with an ultraviolet–visible (UV–Vis) spectrophotometer (TU-1950, Beijing Purkinje General Instrument Co. Ltd, China) in the wavelength range from 350 to 500 nm.

The equilibrium dialysis experiment was carried out using a dialysis bag with a cut-off MW of 30 000 Da in a water bath shaker (37°C, 50 rpm). The solution inside the dialysis bag was 0.1 mol/l NaNO_3_ solution containing 40.0 g/L BSA, and that outside the dialysis bag was 0.1 mol/l NaNO_3_ solution containing 10 μmol/l Fe^3+^ and 0.1 mmol/l citrate. The above solution was sterilized with a 0.22-μm filter; the dialysis bag was sterilized with 75% ethanol; the experimental apparatus, such as beaker, was sterilized with autoclave. The pH of the above solution was adjusted to 7.35–7.45.

In our experiments, the average concentration of iron ions in Hank’s solution containing 40.0 g/l BSA after immersing the iron sheet was nearly 0.5 mmol/l, which was less than 0.6 mmol/l, the molar concentration of BSA. It was reasonable to consider that each albumin might bind only one ferric ion. We set the initial BSA concentration as [BSA]_0_, the free Fe^3+^ concentration at equilibrium as *s* and the average number of Fe^3+^ bound per molecule of BSA at equilibrium as *r.* The balance of BSA binding Fe^3+^ is described as

(1) $$\begin{array}{ccccc} \text{BSA} & + & {\text{Fe}}^{3+} & ⇋ & \text{Fe}-\text{BSA}\\ {\left[\text{BSA}\right]}_{0}-r{\left[\text{BSA}\right]}_{0} & & s & & r{\left[\text{BSA}\right]}_{0} \end{array}$$

And the apparent association constant of Fe–BSA is written as

(2) $$K=r{\left[\text{BSA}\right]}_{0}/[s([\text{BSA}]{}_{0}\;-\;r[\text{BSA}]{}_{0})]=r/\left[s\left(1\;-\;r\right)\right]$$

To calculate this constant, *r* and *s* must be known. The value of *r* could be calculated by the iron concentration outside the dialysis bag, *w*_0_ before dialysis and *w* after dialysis. However, Fe^3+^ can hydrolyze into ferric hydroxide colloids, complicating the composition, and thus *s* is difficult to be quantified. In order to solve this problem, the chelating agent of Fe^3+^, such as citrate with the known stability constants, was introduced. We designated the citric acid as H_3_Ci(OH), and its ionized forms as HCi(OH)^2−^ and Ci(OH)^3−^ near pH 7. If the formation of ternary complex was ignored, we considered only the following reactions:

(3) $$\begin{array}{ccccccc} \text{HCi}{\left(\text{OH}\right)}^{2-} & + & {\;\text{Fe}}^{3+} & ⇋ & {\text{FeCiO}}^{-} & + & {2H}^{+}\\ c_{1} & & s & & c_{3} & & h \end{array}$$

(4) $$\begin{array}{ccccccc} \text{Ci}{\left(\text{OH}\right)}^{3-} & + & {\text{Fe}}^{3+} & ⇋ & {\text{FeCiO}}^{-} & + & H^{+}\\ c_{2} & & s & & c_{3} & & h \end{array}$$

Here the concentrations are denoted below each chemical. The corresponding equilibrium constants are written as *K’* = *c*_3_*h*^2^/(*c*_1_*s*) and *K’’* = *c*_3_*h*/(*c*_2_*s*).

It can be figured out that *K’* = *K’’K*_HCi(OH)_, where *K*_HCi(OH)_ is the acid dissociation constant of the third carboxyl group in citric acid. If the total concentration of citrate is designated as *C* and that of non-protein-bound iron as *w*, we get that *C* = *c*_1_ + *c*_2_ + *c*_3_ and *w *=* s* + *c*_3_ by the law of conservation of mass. Since *s* is much smaller than *c*_3_ under the condition used, *w* ≈ *c*_3_. After combining the following formula, *K’* = *c*_3_*h*^2^/(*c*_1_*s*), *K’’* = *c*_3_*h*/(*c*_2_*s*), *C* = *c*_1_ + *c*_2_ + *c*_3_ and *w* ≈ *c*_3_, the free Fe^3+^ concentration is expressed as

(5) $$s=wh\left(h+K_{\text{HCi}(\text{OH})}\right)/\left[K’’K_{\text{HCi}(\text{OH})}\left(C\;-\;w\right)\right]$$

In our experiments, we detected the quantity *w* from the iron concentration outside the dialysis bag after dialysis, and the quantity *h* from the equilibrium pH. As is known, *K*_HCi(OH)_ = 1.514 × 10^−6^ and *K’’* = 3.090 × 10^9^ in 0.1 mol/l NaNO_3_ solution [40]. It is thus available to calculate the concentration of the free ferric ion with Equation (5) and further the association constant with Equation (2).

### Culture of RAW264.7 cells and Prussian blue staining

RAW264.7 cells (TCM13, Chinese Academy of Science) were cultured in high-glucose Dulbecco’s modified Eagle medium (DMEM, 11995065, Gibco, USA) with 10% fetal bovine serum (Gibco, USA), penicillin (100 U/ml, Gibco, USA) and streptomycin (100 μg/ml, Gibco, USA).

Prussian blue staining was applied to visualize the intracellular iron. RAW 264.7 cells were seeded at a density of 3.2 × 10^5^/well in six-well plates. After reaching a high cell density, the culture medium was replaced by fresh DMEM containing iron compounds for 3 or 12 h. Cells cultured without exogenous iron compounds were set as the negative control, and those cultured with ammonium ferric citrate were set as the positive control. α-FeOOH and α-Fe_2_O_3_ were the insoluble corrosion products of iron sheets immersed in Hank’s solution, and Fe–BSA was the soluble corrosion product of iron sheets immersed in Hank’s solution with BSA. The final concentration of iron in cell culture medium mixed with different iron compounds were 25 μM. After 3 or 12 h, the cells were rinsed with PBS and then fixed in 4% polyformaldehyde at 37°C for 10–20 min. The cells were stained with Prussian blue iron stain kit (G1426, Beijing Solarbio Science & Technology Co. Ltd) following the instruction. The morphology of the cells was observed with an optical microscope (Observer 7, ZEISS, Germany).

ICP-AES was applied to quantify the intracellular iron content. The RAW 264.7 cells were treated with the same procedures as above. After being cultured with iron compounds for 3 or 12 h, the cells were rinsed with PBS and then went through four freeze–thaw cycles to break the cells. The resulting solution was tested by ICP-AES.

### Electrochemical tests

The electrochemical tests were carried out in 300 ml test solution at 37°C using an electrochemical workstation (CHI760E, Shanghai Chenhua, China). A three-electrode system was used with a saturated calomel electrode as the reference electrode, an iron electrode as the working electrode and a platinum electrode as the counter electrode. The test solution included Hank’s solution with and without 40.0 g/l BSA, and the D-Ca/P Hank’s solution with and without 40.0 g/l BSA.

Before the potentiodynamic scanning tests, the immersed iron electrodes were tested for 10 min to obtain a steady open-circuit potential (OCP). Then the potential was scanned from the value of OCP minus 400 mV to the value of OCP plus 400 mV with a scanning rate of 0.33 mV/s. The corrosion current densities of the samples were calculated by Tafel curve fitting with the software equipped in the electrochemical workstation.

The electrochemical impedance spectroscopy (EIS) tests of the iron electrodes in the immersion experiments were performed at OCP with a perturbation amplitude of 20 mV. The applied frequencies were scanned from 100 kHz to 0.01 Hz. The EIS spectra of the above samples were analyzed by Nyquist and Bode plots.

### Atomic force microscope imaging, X-ray photoelectron spectroscopy and micro-bicinchoninic acid assay to detect the adsorbed albumin

An atomic force microscope (AFM) instrument (multimode 8, Bruker, USA) equipped with a J scanner with a maximum range of 125 μm × 125 μm × 5 μm was applied for imaging. The iron sheets and 316L stainless steel sheets used here were further polished with 3.5-μm-diameter diamond suspension and 50-nm-diameter colloidal silica suspension. All the samples and tools were cleaned with ultrapure isopropanol and ethanol, and all test solutions were prepared with ultrapure water and analytical-grade reagents. The iron sheets were immersed for 10 min to minimize the effects of corrosion products. After washing, the iron sheets and the 316L stainless steel sheets were imaged in air by a ScanAsyst-Air probe with a tip radius of 2 nm and a nominal spring constant of 0.4 N/m (Bruker).

For imaging in fluid, the iron sheets were glued to the AFM support by using epoxy glue (Araldite Rapid). The one side of a Teflon disk was glued to the AFM support with Teflon glue (Loctite 770/406), and the other side was glued to a mica sheet by epoxy glue. Ni-mica was prepared by adding 50 μL NiCl_2_ (10 mM, pH 4.0) to the freshly cleaved mica, incubated for 1 h and washed with ultrapure water. For *in situ* imaging, the iron sheets, mica and Ni-mica were directly in contact with the test solution without washing. ScanAsyst-Fluid+ probe with a tip radius of 2 nm and a nominal spring constant of 0.7 N/m (Bruker) was applied for imaging of iron sheets, and PeakFore-HIRS-F-A probe with a tip radius of 1 nm and a nominal spring constant 0.35 N/m (Bruker) were for those of mica and Ni-mica. The height information of every sample was recorded in the PeakForce QNM mode according to the published protocol [41, 42]. NanoScope Analysis software was applied to process and analyze the data.

The elemental composition of the immersed metal surface was characterized with an X-ray photoelectron spectrometer (5000C ESCE system, PHI, Japan). The iron sheets immersed in Hank’s solution with and without 40.0 g/L BSA for 24 h were under an Al-Kα X-ray source radiation with a scanning scale ranging 0–1100 eV. All binding energies referred to the peak of C1s neutral carbon at 284.6 eV. The spectra were deconvolved using the Augerscan software to quantify the element composition.

The unsealed iron sheets were immersed in 10 ml Hank's solution and a series of high-calcium Hank’s solutions containing BSA of different concentrations in a water bath shaker (37°C, 50 rpm). These sheets were not sealed with silicone to avoid the effect of silicone on protein adsorption. After 24 h, the iron sheets were rinsed in Milli-Q water for four times (5 s for each time) to remove non-adherent albumin. Then the iron sheets were incubated in 1 ml of 2% sodium dodecyl sulfate solution (SDS) at 37°C for 1 h to extract adsorbed albumin [43]. The SDS solution also contained CaCl_2_ (0.14 g/l), Na_2_HPO_4_·12H_2_O (0.12 g/l), KH_2_PO_4_ (0.06 g/l), NaHCO_3_ (0.35 g/l) and NaCl (8.0 g/l), in order to inhibit iron corrosion and avoid the interference to the subsequent quantification. The incubated SDS solution was centrifuged (8000 rpm, 5 min), then the concentration of albumin in the supernatant was quantified using the micro-bicinchoninic acid (BCA) assay (Sigma) following the instruction of the manufacturer. The standard solutions in this assay were also prepared by the above SDS solution. The absorbance of the supernatant and the standard solutions was measured in a microplate reader (ELx808, BioTek) at 562 nm. The amount of adsorbed albumin was calculated by subtracting the supernatant absorbance of the iron sheets immersed in Hank’s solution.

### Calculation and determination of the concentration of free Ca^2+^ under given BSA concentration and total Ca^2+^ and calculation of the concentration of total Ca^2+^ under given BSA concentration and free Ca^2+^

The concentration of Ca^2+^ in Hank’s solution was 1.26 mmol/l, and that of 40.0 g/l albumin was nearly 0.6 mmol/l. So the molar ratio of Ca^2+^ to albumin was nearly 2.1 in Hank’s solution with the addition of albumin of the physiological concentration in serum. If one albumin macromolecule binds up to three Ca^2+^, the main chemical balances can be written as follows:

(6) $$\begin{array}{ccccc} \text{BSA} & + & {\text{Ca}}^{2+} & ⇋ & \text{Ca}-\text{BSA}\\ a_{\text{free}} & & b_{\text{free}} & & a_{1} \end{array}$$

(7) $$\begin{array}{ccccc} \text{Ca}-\text{BSA} & + & {\text{Ca}}^{2+} & ⇋ & {\text{Ca}}_{2}-\text{BSA}\\ a_{1} & & b_{\text{free}} & & a_{2} \end{array}$$

(8) $$\begin{array}{ccccc} {\text{Ca}}_{2}-\text{BSA} & + & {\text{Ca}}^{2+} & ⇋ & {\text{Ca}}_{3}-\text{BSA}\\ a_{2} & & b_{\text{free}} & & a_{3} \end{array}$$

Here, *a_i_* and *b*_free_ denote the corresponding concentrations. By the law of conservation of mass, the total concentrations of BSA and Ca^2+^ are equal to the summation of the concentrations of each component,

(9) $$a=a_{\text{free}}\;+\;a_{1}\;+\;a_{2}\;+\;a_{3}$$

(10) $$b=b_{\text{free}}\;+\;a_{1}\;+\;2a_{2}\;+\;3a_{3}$$

The corresponding three equilibrium constants are written as

(11) $$K_{1}=a_{1}/\left(a_{\text{free}}b_{\text{free}}\right)$$

(12) $$K_{2}=a_{2}/\left(a_{1}b_{\text{free}}\right)$$

(13) $$K_{3}=a_{3}/\left(a_{2}b_{\text{free}}\right)$$

The equilibrium constants are known as *K*_1_ = 1513 l/mol, *K*_2_ = 647 l/mol, *K*_3_ = 507 l/mol [44]. According to our experiments, we set *a* = 0.6 mmol/l and *b *=* *1.26 mmol/l. After combining Equations (9)–(13), we figure out that the free Ca^2+^ concentration was 0.70 mmol/l.

The concentration of free Ca^2+^ in the high-calcium Hank’s solution in the presence of 40.0 g/l BSA was controlled at 1.26 mmol/l, which is equal to the total Ca^2+^ concentration in the conventional Hank’s solution free of any protein. We then calculated the total concentration of calcium under a given *b*_free_. Under the condition of high-calcium Hank’s solution, we reasonably assumed that one albumin macromolecule binds up to four Ca^2+^. The binding of the fourth calcium ion is expressed as

(14) $$\begin{array}{ccc} {\text{Ca}}_{3}-\text{BSA}\;\;\;\;+ & {\text{Ca}}^{2+}⇋ & {\text{Ca}}_{4}\;-\;\text{BSA}\\ a_{3} & b_{\text{free}} & a_{4} \end{array}$$

And thus, the fourth equilibrium constant should be taken into consideration,

(15) $$K_{4}=a_{4}/\left(a_{3}b_{\text{free}}\right)$$

For the high-calcium Hank’s solution in the presence of BSA,

(16) $$a=a_{\text{free}}\;+\;a_{1}\;+\;a_{2}\;+\;a_{3}\;+\;a_{4}$$

(17) $$b=b_{\text{free}}\;+\;a_{1}\;+\;2a_{2}\;+\;3a_{3}\;+\;4a_{4}$$

The four equilibrium constants are known as *K*_1_ = 1513 l/mol, *K*_2_ = 647 l/mol, *K*_3_ = 507 l/mol, *K*_4_ = 58 l/mol [44]. For the high-calcium Hank’s solution with 40.0 g/l BSA added, we set *a *=* *0.6 mmol/l, *b*_free_ = 1.26 mmol/l. So the combination of Equations (11)–(13) and (15)–(17) led to the total Ca^2+^ concentration *b *=* *2.16 mmol/l. Similarly, we could figure out that for the high-calcium Hank’s solution adding 8.0 g/l BSA, *a* = 0.12 mmol/l, and for the high-calcium Hank’s solution with 1.6 g/l BSA, *a* = 0.024 mmol/l; then the total Ca^2+^ concentrations were predicted as 1.44 and 1.30 mmol/l, respectively.

For quantitative determination of free Ca^2+^, a series of Hank’s solutions with BSA of different concentrations were incubated in the water bath at 37°C for 10 min, then ultrafiltered by a centrifugal filter device (Ultra-15, Amicon, Millipore, USA) with a cut-off MW of 30 000 Da at 900 *g* for 3 min. The ultrafiltration process was applied for the separation of albumin-bound Ca^2+^ and albumin-free fractions to avoid interference to the subsequent colorimetry [45]. These ultrafiltrates were mixed with murexide, and the final concentration was 0.03 mM. Then the spectra were measured with a UV–Vis spectrophotometer (TU-1950, Beijing Purkinje General Instrument Co. Ltd, China). The working Ca^2+^ standard solutions of concentrations 0.25, 0.5, 1, 2.5 and 5 mM were prepared by the normal saline containing 0.01 M Tris to make the ionic strength and pH equal to the sample. The extinction ratios, the absorbance at 480 to 520 nm were calculated for both standards and samples. The extinction ratios were plotted as a function of Ca^2+^ concentration to make a standard curve.

### Quantification of the calcium and phosphorus adsorbed on the iron sheets

The unsealed iron sheets were immersed in 10 ml Hank's solution with and without 40.0 g/l BSA in a water bath shaker (37°C, 50 rpm). These iron sheets were not sealed with silicone in order to avoid the effect of calcium carbonate in silicone on calcium quantification. After being immersed for 1 or 24 h, the iron sheets were rinsed in Milli-Q water to remove residual test solution on the surface, then were ultrasonically cleaned in nitric acid (5 wt.%) for 20 s to dissolve adsorbed calcium and phosphorus. The mass of calcium or phosphorus was quantified based on the volume and the calcium or phosphorus concentration of the above nitric acid solution measured by ICP-AES.

### Statistical analysis

All data were expressed as mean ± standard deviation. Student’s *t*-tests were applied to assess a significant difference, which was expressed as “*” (*P *<* *0.05), “**” (*P *<* *0.01) or “***” (*P *<* *0.001).

## Results

### Medium albumin enhances the solution of corrosion products of iron in Hank’s solution

BSA was employed as the model biomacromolecule in blood. The corresponding characterizations are shown in Supplementary Figures S2 and S3 and Supplementary Table S2. Hank’s solution was chosen as the main medium, since its inorganic salts, pH and osmotic balance are similar to those of the serum [46]. From the optical photographs of the corroded iron sheets and the SEM images (Supplementary Figure S4), the presence of BSA in the corrosion medium increased the area of the corrosion pits. The corrosion coverage of “Hank’s + BSA” group was nearly 10 times than that of “Hank’s” group (Supplementary Figure S5).

**Figure 2.**
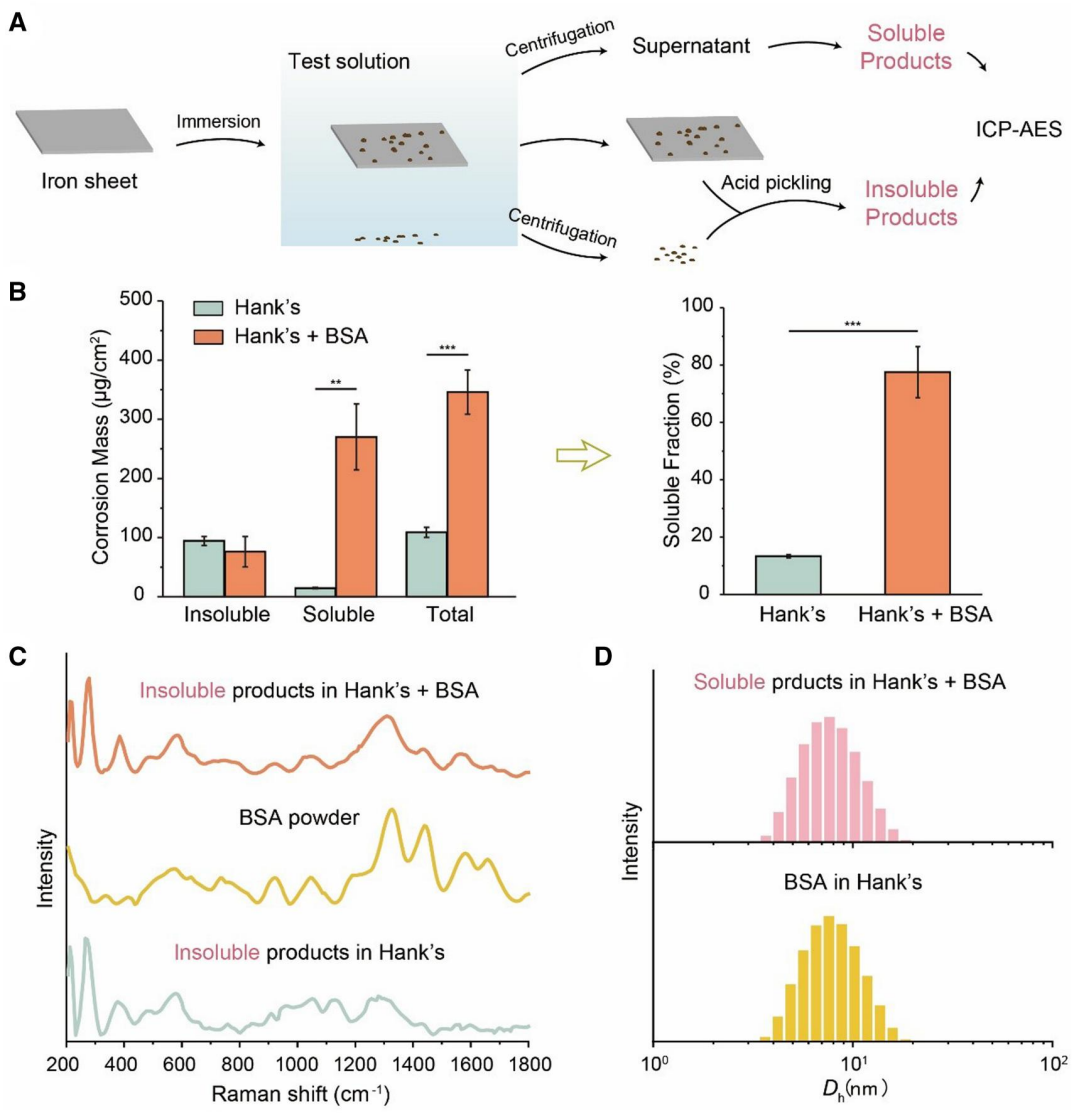
Characterization of the effect of albumin on solubility of corrosion products. The schematic diagram (**A**) and the results (**B**) for determining the mass of insoluble, soluble and total corrosion products, and the soluble fraction of iron sheets after being immersed in Hank’s solution with and without BSA of 40.0 g/L, a physiological concentration in mammal blood, for 24 h. (**C**) Raman spectra of BSA powder and insoluble corrosion products on iron sheets after being immersed in Hank’s solution with and without 40.0 g/L BSA for 24 h. (**D**) Particle size distribution of Hank’s solution containing BSA before and after immersing iron sheets for 24 h characterized by DLS. Prior to testing, the solution was filtered through a 0.22-μm filter (**P <* *0.01; ***P <* *0.001; *n *=* *4).

Corrosion products are divided into two types, insoluble one and soluble one. We separated them with centrifugation and then quantified the amounts of these two parts (Figure 2A). Both the amount and the fraction of soluble corrosion products in “Hank’s + BSA” group were larger than those in “Hank’s” group (Figure 2B), indicating that albumin could improve the solubility of corrosion products to a large extent.

The Raman spectra of iron sheets immersed in Hank’s solution with and without BSA showed the peak of 220, 280, 390 and 1310 cm^−1^ (Figure 2C), which may be attributed to goethite and hematite, namely, α-FeOOH and α-Fe_2_O_3_ [47, 48]. The results showed that albumin had no effect on the structure of insoluble corrosion products.

Soluble iron corrosion products may exist in two forms, one as ions, such as ferric and ferrous ions, and the other as nanoparticles, which could be distinguished by DLS. There was no difference in the particle size distribution between the supernatant in the “Hank’s + BSA” group containing the soluble corrosion products, and the initial Hank’s solution containing BSA prior to immersing (Figure 2D). Hence, the soluble corrosion products were ions complexed by BSA rather than relatively large nanoparticles, otherwise there would be a new size distribution or the size distribution of BSA would be changed.

**Figure 3.**
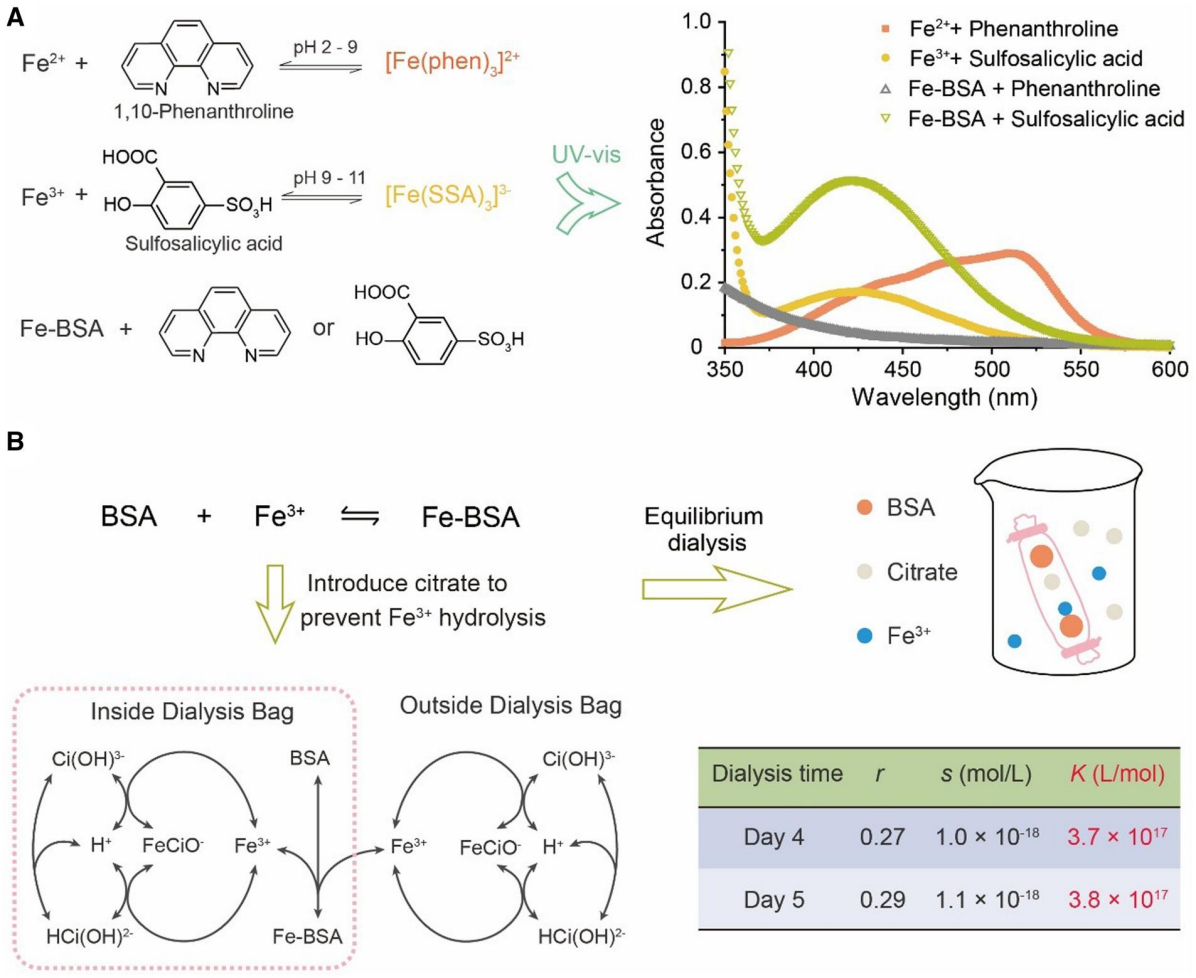
Exploration of the principle that albumin increases the solubility of corrosion products. (**A**) Schematic diagram (left) and result (right) for determining the valence states of iron ions in the Fe-BSA using UV–Vis spectroscopy and metal indicators. (**B**) Schematic illustration of equilibrium dialysis method to determine the apparent association constant of Fe-BSA and main balances that existed inside and outside the dialysis bag. The table lists the key results of the dialysis experiment, which has been in equilibrium after 4 days. Here, *r* is the average number of Fe^3+^ bound per molecule of BSA, and *s* is the free Fe^3+^ concentration at equilibrium.

We employed sulfosalicylic acid with a characteristic peak of 425 nm as the indicator of Fe^3+^, and 1,10-phenanthroline with a peak of 510 nm upon binding as that of Fe^2+^ (Figure 3A). There was only a peak at 425 nm after Fe–BSA was mixed with the indicator, illustrating that the iron ion of the Fe–BSA complex was Fe^3+^ instead of neither Fe^2+^ nor a mixture. It is, however, not trivial to quantify the association constant due to the interference of Fe^3+^ hydrolysis. We eventually employed citrate to chelate the Fe^3+^ based on equilibrium dialysis with the principle shown in Figure 3B and with the calculation approach described in the section of “materials and methods”. The calculated apparent association constant between albumin and Fe^3+^ turned out to be close to 3.8 × 10^17^ l/mol, which agrees with the molecular dynamics and quantum mechanics study [49]. The association constants between Fe^3+^ and transferrin, Fe^3+^ and ethylenediamine-*N*, *N*, *N*′, *N*′-tetraacetic acid (EDTA), Fe^3+^ and glycine, and Fe^3+^ and lactic acid read 4.7 × 10^20^, 1.7 × 10^24^, 1.0 × 10^10^ and 1.3 × 10^7^, respectively [50, 51]. Albumin has a relatively strong ability to bind Fe^3+^ compared with other common chelating agents.

### The soluble Fe–BSA complex can be removed by endocytosis of macrophages

**Figure 4.**
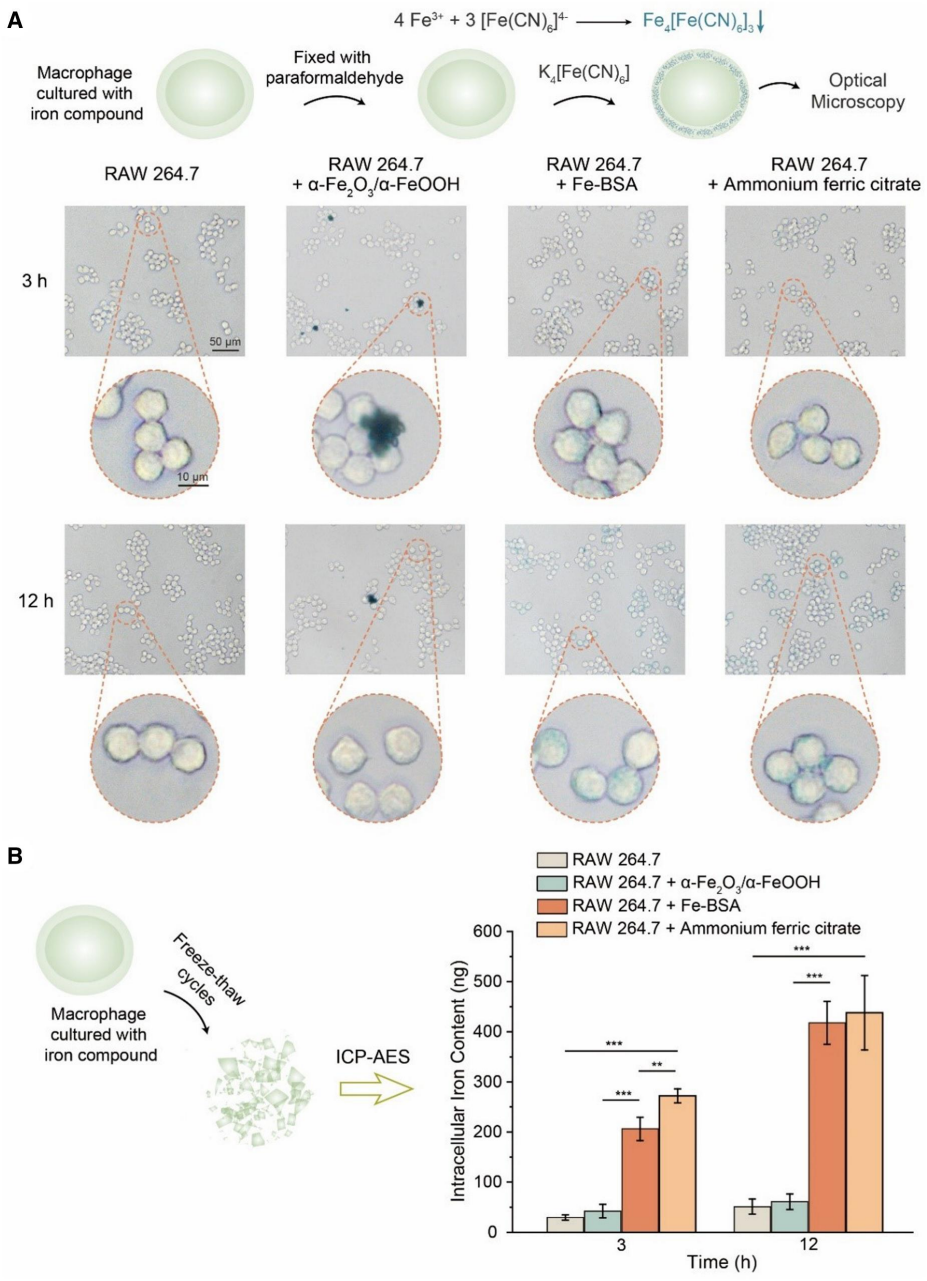
Characterization of endocytosis content of Fe-BSA coordination complex by macrophages. (**A**) Schematic diagram and images of the Prussian blue staining. RAW264.7 cells were cultured with different iron compounds for 3 and 12 h. In the group of RAW264.7 cells cultured with α-Fe_2_O_3_ and α-FeOOH, the bulk blue particles were extracellular and distinct from intracellular iron. (**B**) Schematic diagram and the result of quantifying the iron content in RAW264.7 cells cultured with different iron compounds by ICP-AES. RAW264.7 cells cultured without exogenous iron compounds were set as the negative control, and cultured with ammonium ferric citrate were set as the positive control. α-Fe_2_O_3_ and α-FeOOH were the insoluble corrosion products of iron sheets immersed in Hank’s solution for 24 h. The final concentration of iron in cell culture medium mixed with different iron compounds were 25 μM (**P <* *0.01; ***P <* *0.001; *n *=* *4).

A macrophage cell line RAW264.7 was employed to examine the potential way of clearance of the complex of ferric ion and albumin by cells, since macrophages play a major role in the iron metabolism system [52]. Prussian blue staining was applied to visualize the intracellular iron, since the Fe^3+^ could be coordinated by [Fe(CN)_6_]^4-^ to form Fe_4_[Fe(CN)_6_]_3_, a bluish-green precipitate. From Figure 4A, the RAW264.7 cells cultured with soluble Fe–BSA showed slight bluish-green at 3 h and darker blue at 12 h, which was similar to that cultured with ammonium ferric citrate as the positive control. In contrast, the cells cultured with insoluble corrosion products, α-FeOOH and α-Fe_2_O_3_, showed no bluish-green color, while some bluish-green areas in the images were merely the corrosion products that had not been washed off. The endocytosis amount of the soluble Fe–BSA was far higher than that of α-FeOOH and α-Fe_2_O_3_ (Figure 4B). The above results illustrated that the Fe–BSA, as a soluble corrosion product, could be endocytosed more quickly by macrophages.

Considering the chelating ability of albumin to facilitate the dissolution of insoluble corrosion products, we put forward a viewpoint that albumin may be the transporter to promote the bioabsorption of corrosion products. Some stent implantation experiments have shown no rust particle on the surface of an iron stent when contacting with blood [53]. Now this can be understood from the dissolution of corrosion products promoted by albumin in the blood.

### The enhancement of solubility of corrosion products is universal for other main-stream biodegradable metals and polymer-metal composites in different media

**Figure 5.**
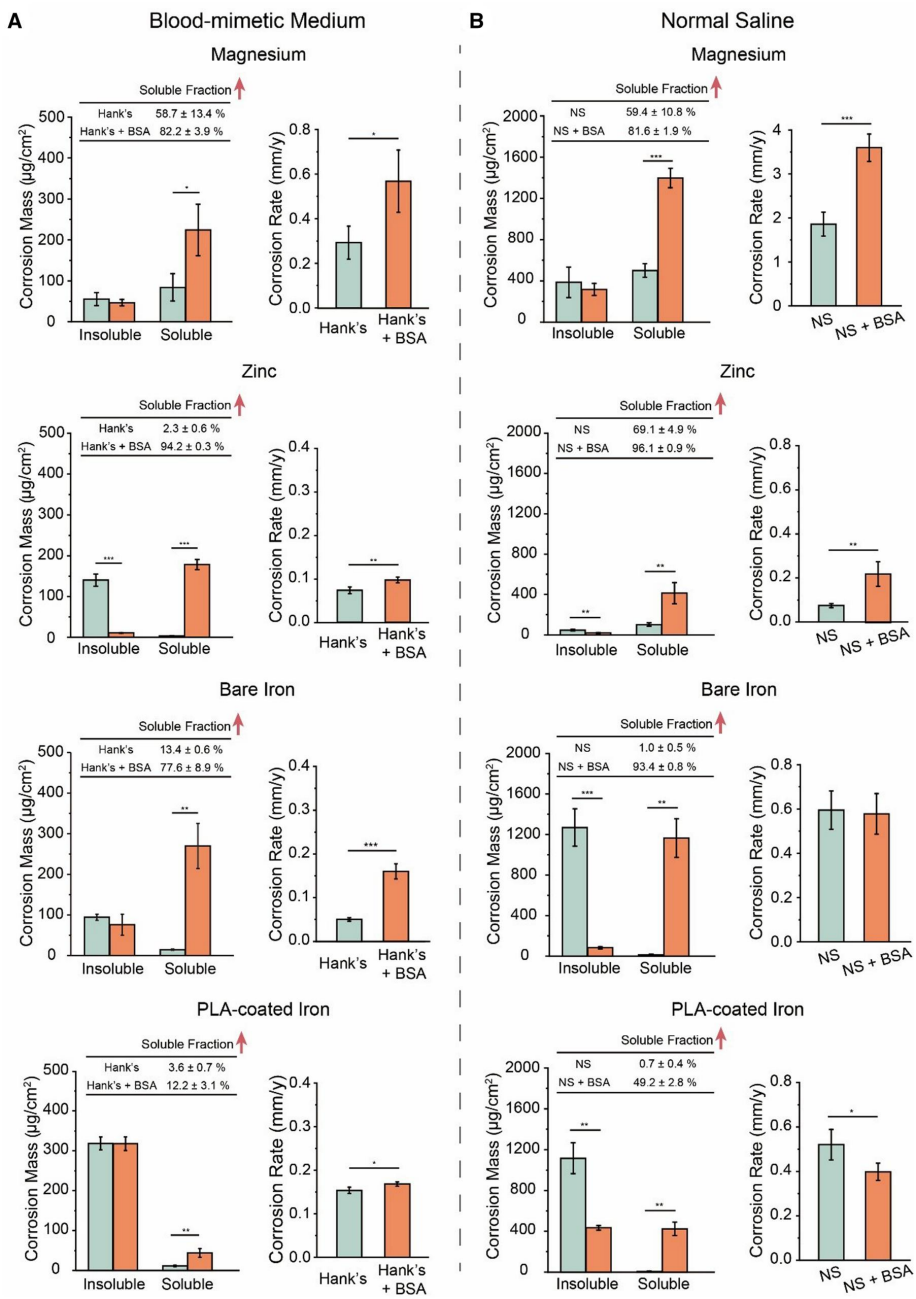
Quantification of the effects of albumin on the solubility of corrosion products and the corrosion rate of biodegradable metals in different biomimetic media. (**A**) The mass of insoluble and soluble corrosion products, and the corrosion rates of magnesium, zinc, bare iron and PLA-coated iron sheets after being immersed in Hank’s solution with and without 40.0 g/L BSA for 24 h. (**B**) Corrosion behaviors in normal saline (NS). the corrosion rate was calculated by the corrosion mass of metal at 24 h and the metal density. The red arrows indicate the increase in soluble fraction (*P <* *0.05; **P <* *0.01; ***P <* *0.001; *n *=* *4).

We further examined magnesium, zinc and PLA-coated iron in Hank’s solution. The PLA coating on iron sheets was obtained via ultrasonic spraying (Supplementary Figure S6). We also examined the effects of proteins in media beyond Hank’s solution. The addition of medium protein resulted in a significant increase in the soluble fraction of the corrosion product for magnesium, zinc, bare iron and PLA-coated iron in Hank’s solution and normal saline (Figure 5), and for bare iron in PBS and D-Ca/P Hank’s solution (Supplementary Figures S7 and S8). These results have illustrated that albumin could improve the solubility of the corrosion products of degradable metals in most biomimetic media and this effect was not blocked by polyester coatings.

### Serum proteins lead to different trends of corrosion rates, dependent upon metal and medium types

We calculated corrosion rates from time dependence of the mass of total corrosion products (Supplementary Figure S9). For magnesium, zinc, bare iron and PLA-coated iron in Hank’s solution, the corrosion rates in the presence of BSA were larger than those without BSA (Figure 5A), illustrating that albumin could accelerate the corrosion of all of these main-stream biodegradable metals in Hank’s solution.

We also examined the effects of BSA in other biomimetic media. According to Figure 5B, BSA had no significant net effect on the corrosion of bare iron in normal saline, but accelerated the corrosion of magnesium and zinc, and slowed down the corrosion of PLA-coated iron. In PBS and D-Ca/P Hank’s solution, BSA inhibited the corrosion of bare iron (Supplementary Figures S7 and S8). Hence, the albumin effects on corrosion rates are dependent upon corrosion media and rather complicated.

### A unified mechanism to explain the effects of serum proteins on the corrosion of biodegradable metals

The bare iron sheets in Hank’s solution in the presence of albumin were applied to clarify the effects of serum proteins. From the EDS mapping images (Supplementary Figure S10) and the semi-quantitative results (Supplementary Table S3), the surfaces of corroded iron immersed in Hank’s solution with and without BSA both contained Ca and P elements, indicating that the deposition of the inorganic salts rich in Ca and P.

**Figure 6.**
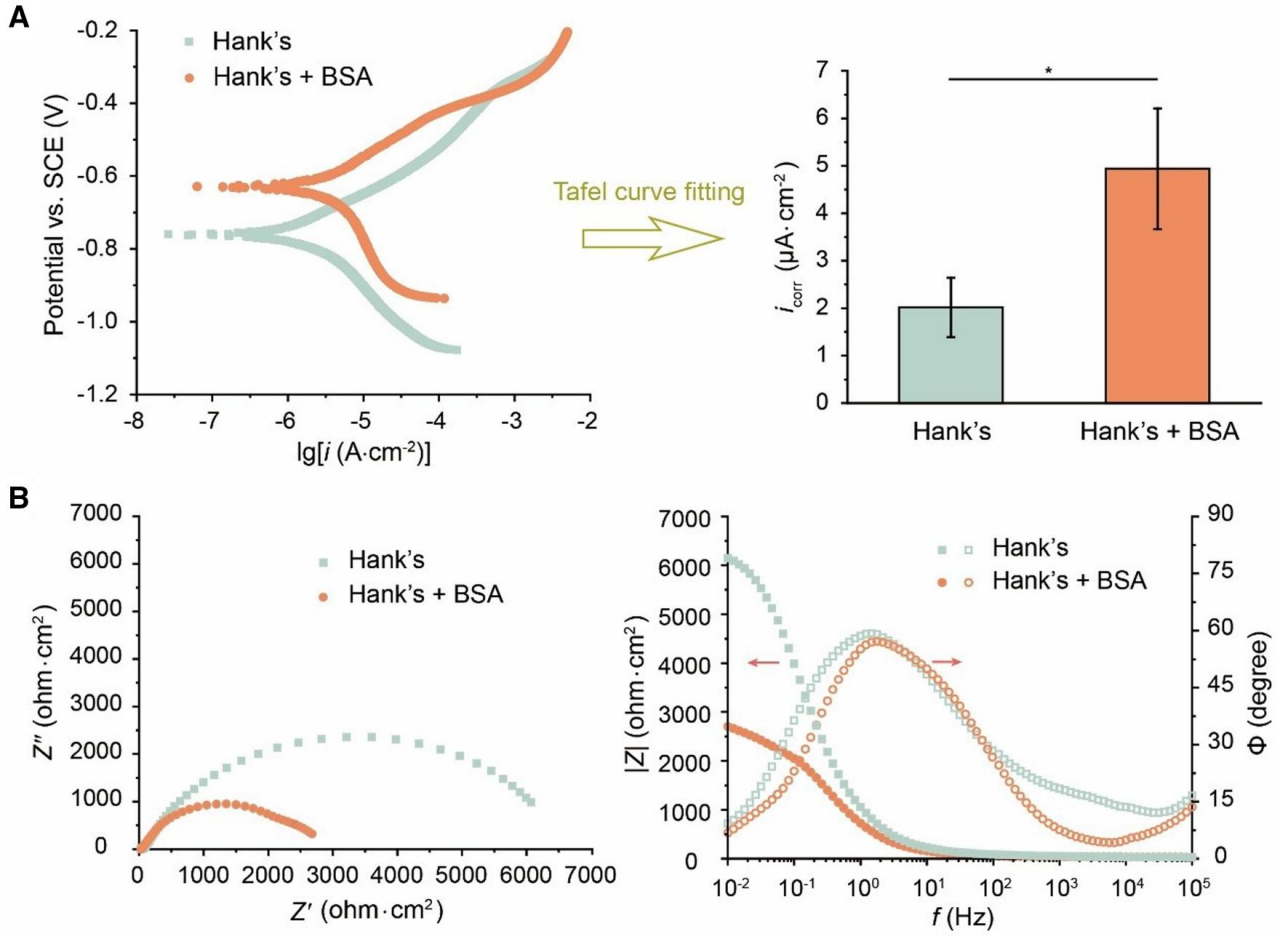
Electrochemical tests of iron electrodes immersed in Hank’s solution with or without BSA. (**A**) Tafel curves (left) and corrosion current density (right) of iron electrodes after being immersed in Hank’s solution with or without 40.0 g/L BSA for 24 h. (**B**) Nyquist plots (left) and Bode plots (right) of iron electrodes after being immersed in Hank’s solution with or without 40.0 g/L BSA for 24 h. *Z’* and *Z’’* represent the real and imaginary parts of the complex impedance *Z*, respectively (*P <* *0.05; *n *=* *4).

We further applied electrochemical tests, such as potentiodynamic polarization curve testing and EIS with the principle shown in Supplementary Figures S11 and S12. By fitting the Tafel curve, the corrosion current density (*i*_corr_) of iron in “Hank’s + BSA” group was nearly 2.5 times than that of “Hank’s” group (Figure 6A), strengthening the acceleration of iron corrosion in the presence of albumin. From the Nyquist plots and Bode plots (Figure 6B), the transfer resistance *R*_t_ in the “Hank’s” group was higher, may be due to the passivation of the deposited salts rich in Ca and P (Supplementary Figure S10). The *R*_t_ in “Hank’s + BSA” group was significantly smaller, implying that the formation of the Ca/P passivation layer was significantly disturbed by albumin.

**Figure 7.**
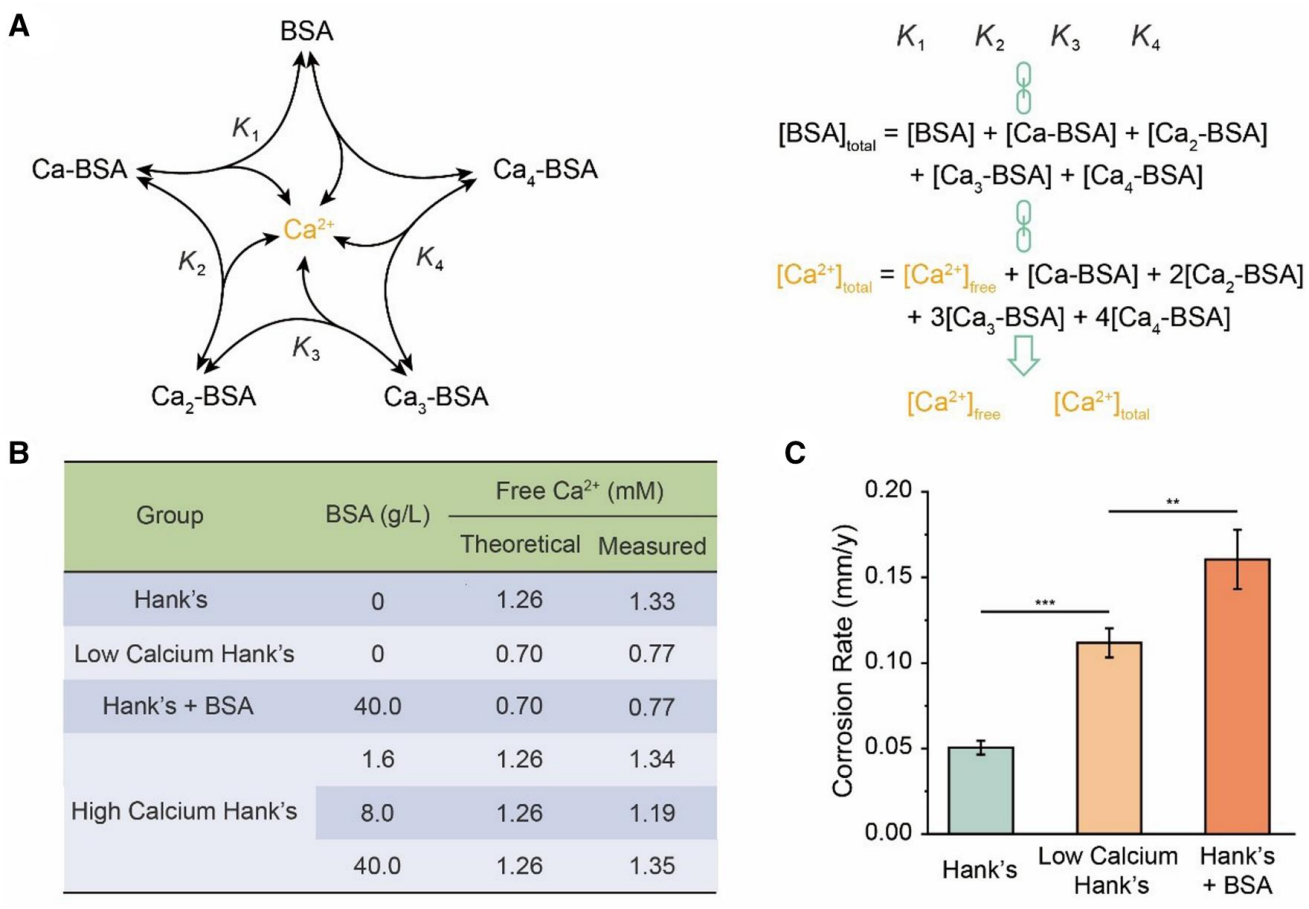
Study on the chelation effect of albumin on iron corrosion. (**A**) Schematic illustration of the main balances between BSA and four Ca^2+^ (left) and the method to calculate the concentration of free or total Ca^2+^ (right) in Hank’s solution in the presence of BSA. (**B**) The contents of free Ca^2+^ in the test solutions. (**C**) Corrosion rate of iron sheets after being immersed in Hank’s solution with BSA and without BSA, and in low calcium Hank’s solution for 24 h (**P <* *0.01; ***P <* *0.001; *n *=* *4).

It was hypothesized that the albumin adjusted the Ca/P layer partially by decreasing the concentration of free Ca^2+^. From Figure 7A, the correlation between the concentration of free Ca^2+^ and total Ca^2+^ could be established by the stepwise association constants between the free Ca^2+^ and BSA, and the conservation of matter. The theoretical calculation in the section of “materials and methods” showed that the free Ca^2+^ in Hank’s solution with 40.0 g/l BSA was 0.70 mM, much lower than 1.26 mM, the free Ca^2+^ concentration in Hank’s solution. We introduced murexide as the Ca^2+^ indicator to confirm our theoretical prediction, with the principle shown in Supplementary Figure S13 and the standard curve shown in Supplementary Figure S14. The measured values in Figure 7B are close to the theoretical values.

In order to examine the influence of lowering the free Ca^2+^ on iron corrosion, we prepared the low calcium Hank’s solution which contained the same free Ca^2+^ as Hank’s solution with 40.0 g/l BSA. From Figure 7C, the corrosion rate of iron in the group of “low calcium Hank’s” was significantly higher than that of “Hank’s” group, confirming the hypothesis that albumin affects the formation of the Ca/P layer by decreasing the free Ca^2+^. Nevertheless, the corrosion rate of iron in the group of “low calcium Hank’s” was still lower than that of “Hank’s + BSA” group, indicating that albumin has another effect.

Surface–macromolecules interactions have a great effect on the properties of the material [54]. It was hypothesized that some albumin was adsorbed on the iron surface and interfered the Ca/P layer, and thus affected the corrosion of iron. From the X-ray photoelectron spectroscopy results, the N1s peak area of corroded iron immersed in Hank’s + BSA solution was larger than that in Hank’s solution (Supplementary Figure S15), so as the fraction of nitrogen (Supplementary Table S4), both confirming that the BSA was adsorbed onto the iron surface.

**Figure 8.**
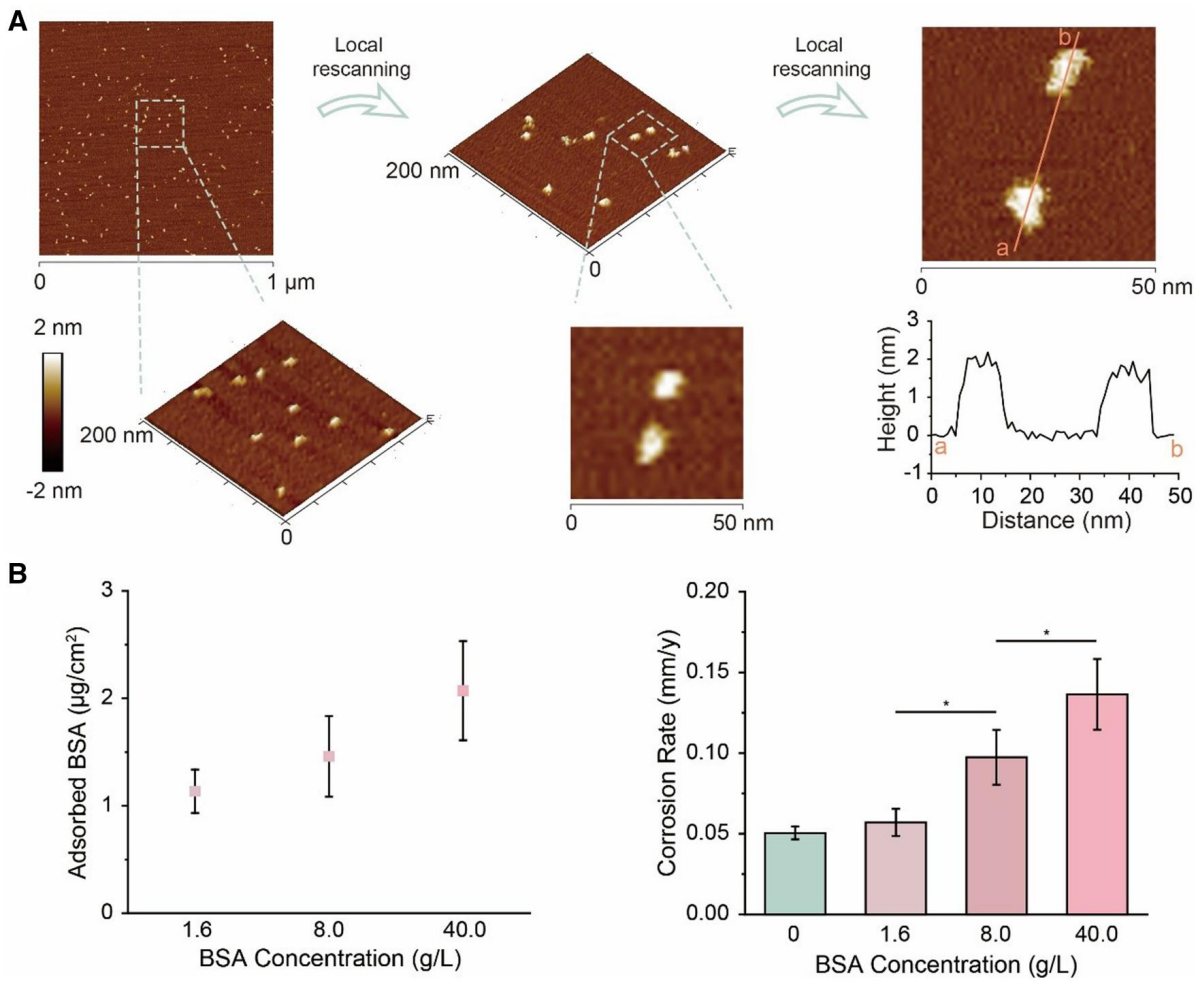
Study on the adsorption effect of albumin on iron corrosion. (**A**) AFM images of BSA on the Ni-mica in water. Images were scanned from the bottom to the top. For high quality BSA imaging, the Ni-mica was rescanned locally to obtain the images of 200 nm × 200 nm and 50 nm × 50 nm, while the other images were the enlargement of the larger-area images. The rescan interval was about 10 min, and the scanning time was about 2.5 min. The *Z* range of 3D images of Ni-mica was from −2 to 2 nm and the *Z*-axis was magnified twice. (**B**) The amount of adsorbed BSA on iron sheets (left) and the corrosion rates of iron sheets (right) after being immersed in high-calcium Hank’s solutions with BSA or Hank’s solution for 24 h. The free Ca^2+^ concentration was kept as 1.26 mM (*P <* *0.05; *n *=* *4).

We also applied AFM to observe the albumin aggregates and even the single molecular albumin on metal and mica in air and in fluid (Supplementary Figures S16–S19). Considering that the probe may be contaminated by albumin during *in situ* imaging in fluid, the sample was rinsed with ultrapure water to remove excess proteins prior to AFM imaging in liquid. For high-resolution imaging, the selected area was zoomed and rescanned (Figure 8A). From the images of 50 nm × 50 nm, the single molecular albumin exhibited the subcircular shape with nearly 10 nm length in the *XY* direction and 2 nm height in the *Z* direction. Compared with the size of crystalized BSA (Supplementary Figure S20), the adsorbed albumin showed a spreading state, which may arise from the interaction of the protein with the solid surface.

Our further calculations (Supplementary Sections S1 and S2) have revealed that the fraction of adsorbed BSA among the total BSA in solution was too low to alter the BSA concentration in medium, and that the adsorbed albumin formed a multilayer protein membrane.

In particular, we designed a series of “high-calcium Hank’s solutions”, which had similar free Ca^2+^ contents despite of varied BSA concentrations (Figure 7B and Supplementary Table S1). The adsorbed BSA was quantified based on the micro-BCA assay with the principle shown in Supplementary Figure S21. The amount of BSA on the iron surface increased with the BSA concentration, so as the corrosion rates (Figure 8B), indicating that the adsorbed albumin accelerates iron corrosion greatly.

We also measured the amount of adsorbed calcium in “Hank’s + BSA” group, and observed no significant difference from that in the “Hank’s” group. The amount of adsorbed phosphorus was even larger (Supplementary Figure S22). This illustrates that the protein adsorption might decrease the dense extent in spite of increasing the total amount of the Ca/P layer.

In order to further examine the relation between the adsorbed BSA and the Ca/P layer, we introduced a D-Ca/P Hank’s solution, which is free of Ca^2+^ and H_2_${\text{PO}}_{4}^{-}$/${\text{HPO}}_{4}^{2-}$ and thus prevents the formation of the Ca/P layer. There was no significant difference of *i*_corr_ in D-Ca/P Hank’s solutions with and without BSA (Supplementary Figure S23A). From Supplementary Figure S23B, the *R*_t_ of the “Hank’s” group was much larger than that of the “D-Ca/P Hank’s” group, indicating the contribution of the Ca/P layer. The contribution of the BSA layer reflected by the difference of *R*_t_ between “D-Ca/P Hank’s” and “D-Ca/P Hank’s + BSA” was much smaller than that of the Ca/P layer, indicating that the adsorbed protein layer was much less compact than the Ca/P layer.

**Figure 9.**
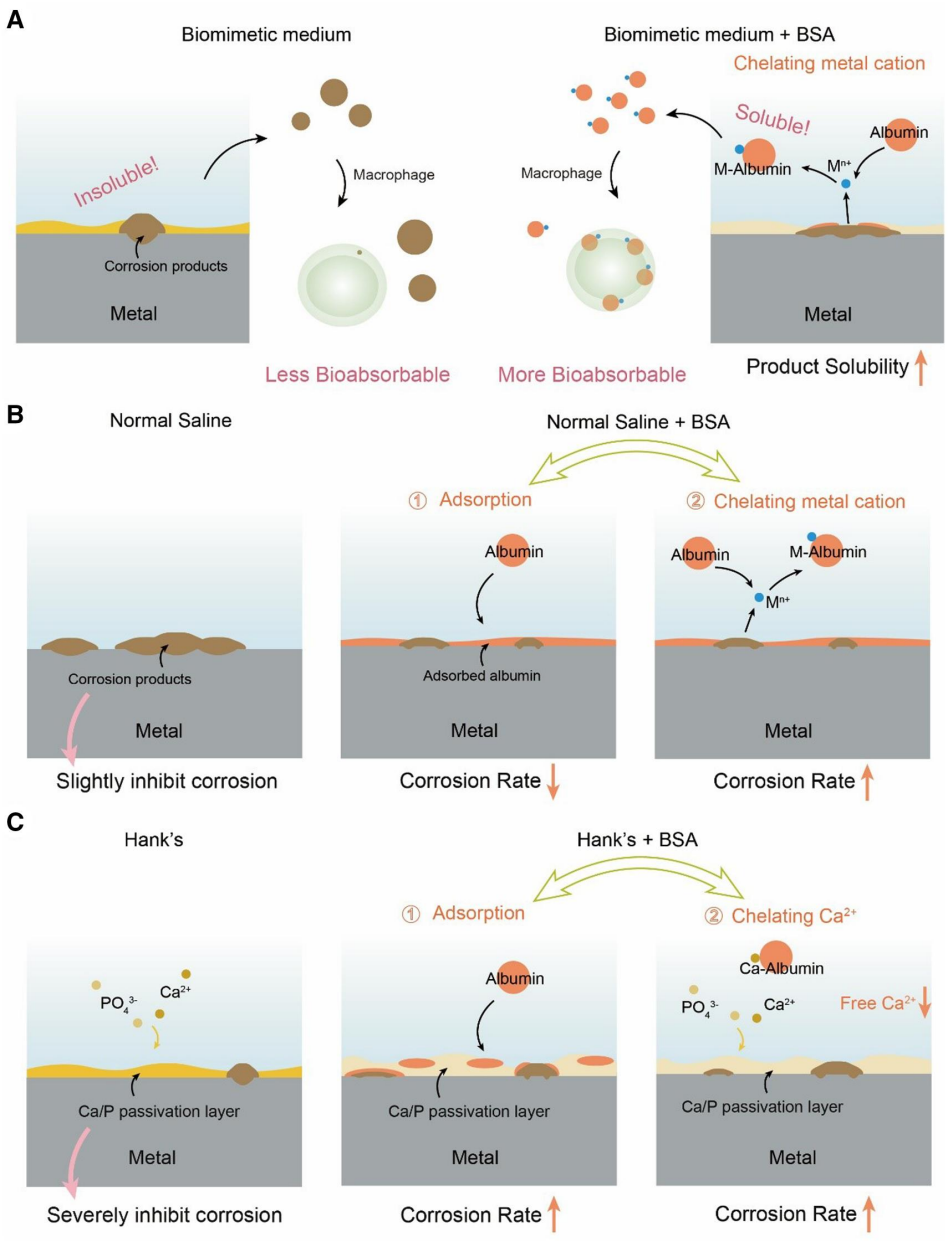
Schematic diagram of the mechanisms accounting for the effects of albumin on solubility of corrosion products and rate of metal corrosion. (**A**) The albumin improves the corrosion products solubility via chelating metal cations. (**B**) In a medium such as normal saline that does not form a Ca/P passivation layer, the albumin inhibits metal corrosion due to the barrier layer of the adsorbed albumin on the solid surface, and meanwhile accelerates metal corrosion due to the chelating effect to metal cations. (**C**) In a medium such as Hank’s solution that probably forms a dense passivation layer, the albumin accelerates iron corrosion via the interference effect of the adsorbed albumin to the Ca/P passivation layer and the chelating effect to decrease the free Ca^2+^ in the corrosion medium.

The mechanisms accounting for the effects of albumin on corrosion of biodegradable metals are schematically presented in Figure 9. The increase in the solubility of corrosion products is accompanied with a shift of precipitation-dissolution equilibrium. Albumin can bind metal ions, thus decrease free metal ions in the equilibrium and promote the dissolution of the precipitate of the corrosion products (Figure 9A). This is reasonable because BSA contains more acidic amino acid residues than alkaline residues, and is of isoelectric point 4.7, and thus negatively charged at physiological pH. The effect of chelation is universal in most biomimetic media.

At the interface of metals and media, the OH^−^ generated by corrosion diffused around, making the local pH higher than the pH in bulk solution. With the increase in local pH, the OH^−^ participated in the formation of corrosion products and was consumed, so the local pH would achieve balance during corrosion (Supplementary Figure S1). In Hank’s solution, the OH^−^ would also participate in the deposition of Ca/P layer and reach balance. Some pH monitoring experiments showed that albumin had little effect on local pH near the interface [37], indicating that the effect of albumin on corrosion rate may be in other ways.

In normal saline, albumin has two opposite “inherent” effects on metal corrosion (Figure 9B): (1) albumin can be adsorbed on the material surface, and the adsorption layer may block the electrochemically active sites thus slow down corrosion and (2) albumin has a strong binding ability with metal cations, which may promote the anodic reactions of metal corrosion and the dissolution of surface corrosion products, both of which can accelerate corrosion (Figure 9B). It is their competition that determines whether the corrosion is accelerated, decelerated or unchanged.

Those “inherent” effects are, however, not predominant in Hank’s solution, where a dense inorganic passivation layer can be formed on the substrate surface. The formation of the Ca/P layer comes from precipitation-dissolution equilibrium, which is influenced by the concentrations of free Ca^2+^, ${\text{PO}}_{4}^{3-}$, and possibly other ions such as OH^−^. Albumin accelerates the corrosion of metal in Hank’s solution owing to its two effects on the Ca/P passivation layer (Figure 9C): (1) albumin with some amino acid residues of carboxylic group etc can chelate Ca^2+^, and the significant decrease in free Ca^2+^ makes the equilibrium associated with the Ca/P layer towards the dissolution based on Le Châtelier’s Principle [55] and (2) the adsorption layers of albumin had a lower transfer resistance compared to the Ca/P layer (Supplementary Figure S23B). The more albumin adsorbed on the surface, the stronger its interference to the default dense Ca/P layer, resulting in the decrease in the overall transfer resistance of the surface and a weakening in the corrosion resistance of the metals. As a result, the puzzling phenomena of metal corrosion in different media in the presence of serum proteins are interpreted by a unified mechanism.

## Discussion

Medical devices are widely used in the treatment of diseases [1, 56–59]. Biodegradable metals and biodegradable polymers have recently been paid much attention in the research and development of next-generation medical devices and drug delivery carriers [5, 60–66]. The underlying fundamental questions are fascinating, such as cell–material interactions [67–74], biosafety [75–78] and degradation control [79–84]. Iron-based, magnesium-based and zinc-based biodegradable metals have been investigated in cardiovascular stents, orthopedic implants and so on [4, 8, 15]. It is very important to have better understanding of the corrosion behaviors of biodegradable metals and their interactions with body fluids for the successful and safe performance of the implants [85]. The corresponding fundamental research can help us to get a better biomimetic medium, reduce the animal sacrifice and accurately design the system of biodegradable materials.

### The improved Hank’s solution

It is important to accurately predict *in vivo* degradation based on *in vitro* tests. The present study strongly reminds us of necessity to introduce proteins in biomimetic media to access metal corrosion besides pH, osmotic balance and the composition of ions. Taking the vascular environment as an example, we herein propose an improved Hank’s solution, “high-calcium Hank’s + 40.0 g/L BSA” (Supplementary Table S1). The improved Hank’s solution contains a physiological concentration of albumin to consider the effects of accelerating corrosion and increasing solubility of corrosion products. Considering chelating of some Ca^2+^ by albumin, the free Ca^2+^ content is within the range of the physiological concentrations [45]. Since albumin is able to scavenge hydroxyl radicals (OH•) [15], the improved Hank’s solution is a significantly optimized *in vitro* medium, which can not only simulate the metal degradation and bioabsorption of corrosion products but also mimic appropriate oxidative damage to surrounding tissues *in vivo*.

### Potential impact to precision medicine

The precision medicine demands the elucidation of key factors to influence the medicine efficacy for individuals. The study raises a new concern of the blood parameter which is closely related to the dynamic property of a biodegradable material. Albumin concentrations in human serum vary with age and sex, reaching a peak around the age of 20 and then decreasing with age, with a maximum difference of nearly 10 g/l [86]. Hypoalbuminemia, defined by the serum albumin less than 35 g/l, is associated with malnutrition, cirrhosis and nephrotic syndrome, with a prevalence of about 3% in pregnant women [87]. Not only albumin, calcium ion also varies greatly in the population, such as hypercalcemia defined as calcium concentration higher than 10.5 mg/dl, and hypocalcemia defined as calcium concentration less than 8.5 mg/dl. Hypercalcemia is mainly caused by primary hyperparathyroidism or malignancies with a prevalence in the general population up to 0.1% [88]. The most common cause of hypocalcemia is post-surgical hypoparathyroidism with a long-term prevalence of about 2 per 10 000 [89]. The differences in albumin and calcium ion in the population suggest that the effects of albumin and calcium ion should be considered when designing a metal-based biodegradable stent, and a concentration detection prior to implantation is now suggested in order to assist the selection of a biodegradable stent of appropriate parameters to fit the individual patient.

### Potential impact to study of macromolecule-metal interactions and development of next-generation medical devices composed of metal-polymer composite

The present study has highlighted the role of interaction of macromolecules and metals in regulating the rates of degradation and the bioabsorption of the degradation products of corrodible metals. Biodegradable polymers, such as polyesters, are widely used in tissue regenerative medicine, drug delivery systems and other fields [90, 91]. Consistent with the interference of the adsorbed BSA to the Ca/P layer and thus accelerating of iron corrosion, the PLA-coated iron has exhibited quicker corrosion than bare iron in blood mimetic Hank’s solution (Figure 5A), which has resolved a bottleneck of the iron-based implant to deal with cardiovascular diseases as a biodegradable stent of an appropriate degradation rate. The polymer coating is more important at the later stage after endothelialization and then the implant does not contact with serum proteins.

This principle has been applied in metal–polymer (Fe–PLA) composite stents developed by the authors’ group in cooperation with other teams. The corresponding coronary stent with excellent high mechanical properties and appropriate degradation rate is now in clinical trial. The statistics of all clinical cases will be reported by clinical teams in the future.

## Conclusions

This study has reported for the first time that albumin promotes solubility of corrosion products of biodegradable metals in most biomimetic media and revealed that the underlying mechanism is relevant to the strong ability of soluble proteins to complex metal cations. Furthermore, this study strengthened the significant role of albumin in accelerating corrosion of biodegradable metals in blood mimicking Hank’s solution. The key lies in the Ca/P passivation layer, which plays a major role in inhibiting corrosion. The adsorption of albumin interferes the formation of a dense passivation layer and thus increases the corrosion rate. The decrease in free Ca^2+^ in the corrosion medium caused by protein chelation is the other key reason of albumin to speed up metal corrosion in Hank’s solution. The “confusing” phenomena of corrosion in physiological or biomimetic media including Hank’s solution, normal saline and others have been interpreted from the competitive cues in a unified way. The mechanistic insight elucidated in this work enables the regulation of corrosion rates and product bioabsorbability of biodegradable metals.

This work has highlighted the important effects of proteins on corrosion behaviors of biodegradable metals. An improved Hank’ solution with a high total calcium content and a physiological level of macromolecules is recommended as a biomimetic medium to access metal biodegradation in the future. The principle revealed in this article is universal for biodegradable metals, and helpful for accurately evaluating metal corrosion in biomimetic media and correctly designing next-generation biodegradable medical devices in the formulism of precision medicine.

## Supplementary Material

rbad112_Supplementary_Data
