# Supplementary material for: Effects of serum proteins on corrosion rates and product bioabsorbability of biodegradable metals
Source: Regen Biomater. 2023 Dec 12;11:rbad112. doi: 10.1093/rb/rbad112 (PMC10761199; doi:10.1093/rb/rbad112)
Supplement: rbad112_Supplementary_Data [file rbad112_supplementary_data.pdf]

SUPPLEMENTARY INFORMATION

Effects of serum proteins on corrosion rates and product bioabsorbability of biodegradable metals

Hongjie Zhang, Xin Li, Zehua Qu, Wanqian Zhang, Qunsong Wang, Dinglingge Cao, Yaoben Wang, Xin Wang, Yang Wang, Lin Yu, Jiandong Ding*

State Key Laboratory of Molecular Engineering of Polymers, Department of Macromolecular Science, Fudan University, Shanghai 200438, China

* Corresponding author. Email address: jdding1@fudan.edu.cn

The Supporting Information contains supplementary calculations followed by supplementary figures and supplementary tables. The 23 supplementary figures and 4 supplementary tables are shown in sequence mentioned in the main manuscript.

Supplementary Calculations:

S1. Calculation of the Fraction of Adsorbed Bovine Serum Albumin (BSA) among the Total BSA in Solution.

The surface area of iron sheets was 2.0 cm^2 and the volume of the test solution was 10 mL. The fraction of adsorbed BSA in 1.6 g/L BSA was 1.13×2.0/(1.6×10000) = 0.014%, where 1.13 was the amount of adsorbed BSA on the surface of iron as shown in **Figure 8B**. Similarly, the fraction of adsorbed protein in 8.0 g/L BSA was 1.46×2.0/(8.0×10000) = 0.0035%, and that of 40.0 g/L BSA was 2.07×2.0/(40.0×10000) = 0.0010%. The fraction was so low that the adsorption has little effect on the BSA concentration in corrosion medium.

S2. Calculation of the Layer of Adsorbed BSA on the Iron Surface.

We assumed that the arrangement of BSA adsorbed on the surface resembled a crystal. The parameters of the unit cell of the BSA crystal are as follows: lengths a = 21.57 nm, b = 4.51 nm, c = 14.24 nm, angles α = 90°, β = 114.01°, γ = 90°, based on the database in protein data bank (PDB ID: 3V03). Then the volume of unit cell $V = a\times\sin\beta\times b\times c$ = 1265.4 nm^3. Since it is unknown which crystal face contacted the surface, we simply equated the monoclinic unit cell to a cubic unit cell with the length $a' = \sqrt[3]{V}$ = 10.82 nm. Considering that a unit cell contains two BSA molecules, the mass of one layer adsorbed BSA on a surface of 1 cm^2 was $(10^7/10.82)^2/(6.02\times10^{23})\times66700\times2$ = 0.189 μg. The number of adsorbed layers of 1.6 g/L BSA was 1.13/0.189 = 6.0, that of 8.0 g/L BSA was 1.46/0.189 = 7.7, and that of 40.0 g/L BSA was 2.07/0.189 = 11.0, indicating a multilayer protein membrane.

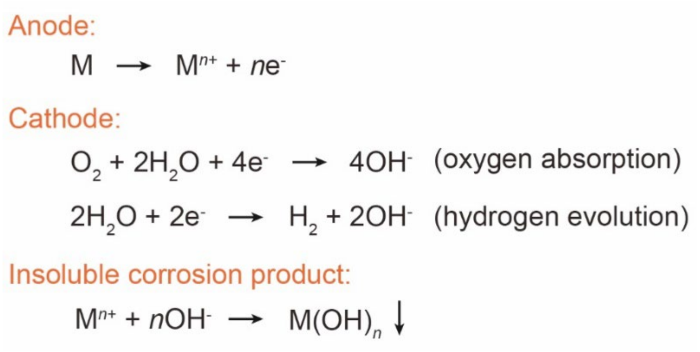

Figure S1. The main reactions of biodegradable metals (M) in the nearly neutral physiological environment. Metal corrosion includes metal dissolution at the anode and hydrogen evolution or oxygen adsorption at the cathode, often accompanied by the formation of insoluble corrosion products. The standard electrode potential (SEP) of a metal determines the type of its cathode reaction. The SEP of magnesium is -2.37 V, and the cathode reaction goes through hydrogen evolution; the SEPs of zinc and iron are -0.76 V and -0.44 V respectively, and their cathodic reactions go through oxygen absorption as usual. The corrosion products are mainly composed of metal hydroxides and oxides, depending on pH, anions, dissolved oxygen and temperature. Iron ions have two valence states, so the corrosion products are more complex — an oxygen-poor environment favours the formation of magnetic magnetite, while an oxygen-rich environment favours that of iron oxyhydroxides.

Table S1. Compositions of Hank's solution, Hank's solution containing 40.0 g/L BSA, D-Ca/P Hank's solution, low calcium Hank's solution and a series of high calcium Hank's solutions.

Composition	Content (g/L)						
	Hank's	Hank's + BSA	D-Ca/P Hank's	Low Calcium Hank's	High Calcium Hank's + 40.0 g/L BSA	High Calcium Hank's + 8.0 g/L BSA	High Calcium Hank's + 1.6 g/L BSA
BSA	-	40.0	-	-	40.0	8.0	1.6
$CaCl_2$	0.140	0.140	-	0.077	0.240	0.160	0.144
KCl	0.40	0.40	0.40	0.40	0.40	0.40	0.40
KH_2PO_4	0.06	0.06	-	0.06	0.06	0.06	0.06
$MgCl_2 \cdot 6H_2O$	0.10	0.10	0.10	0.10	0.10	0.10	0.10
$MgSO_4 \cdot 7H_2O$	0.10	0.10	0.10	0.10	0.10	0.10	0.10
NaCl	8.0	8.0	8.0	8.0	8.0	8.0	8.0
$NaHCO_3$	0.35	0.35	0.35	0.35	0.35	0.35	0.35
$Na_2HPO_4 \cdot 12H_2O$	0.12	0.12	-	0.12	0.12	0.12	0.12
D-glucose	1.0	1.0	1.0	1.0	1.0	1.0	1.0

Note: The colored characters highlight the difference among the media.

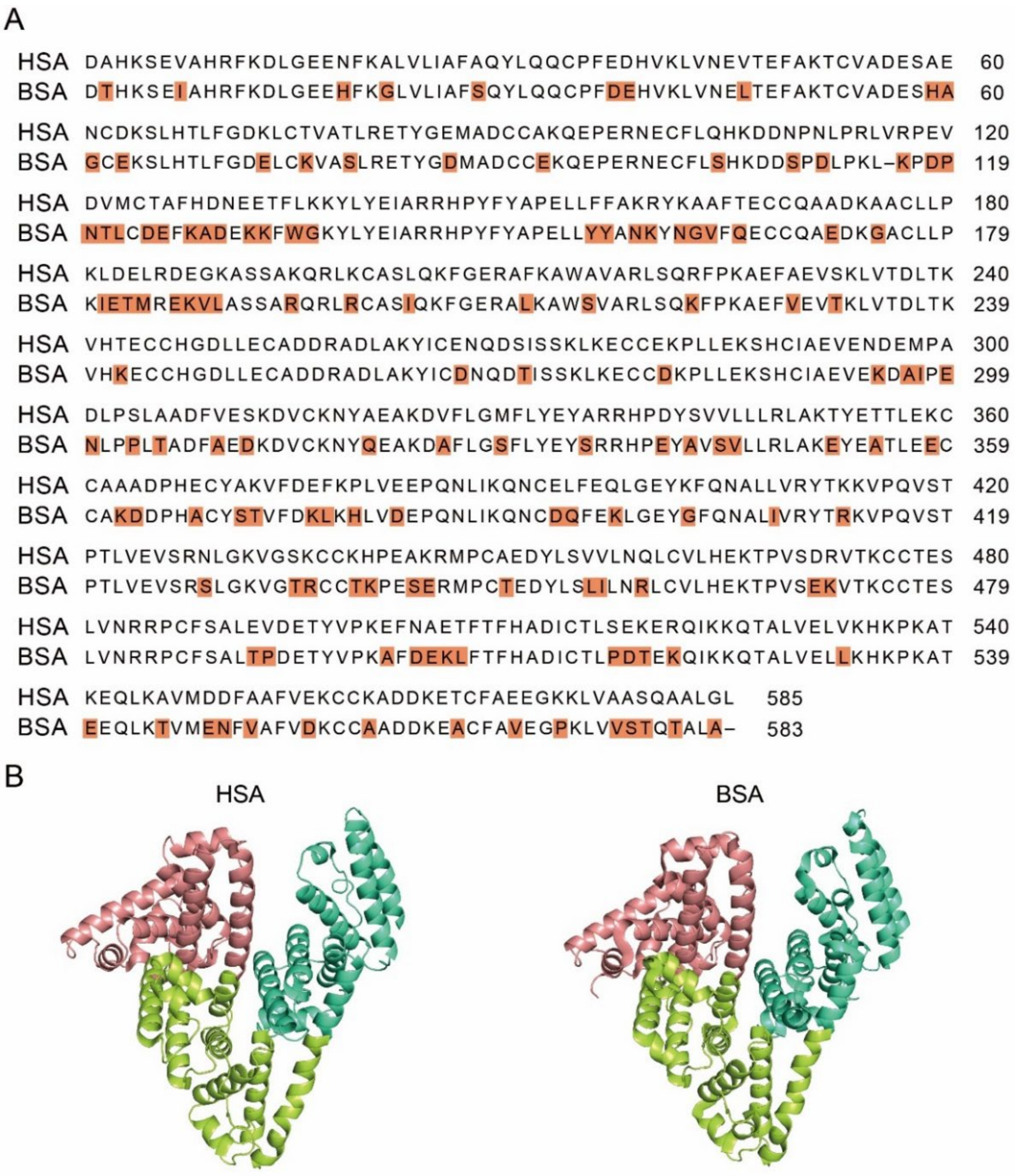

Figure S2. Similarity of BSA to human serum albumin (HSA). (A) The primary structures of HSA and BSA. The different amino acid residues are identified in orange color. G, Glycine; A, Alanine; V, Valine; L, Leucine; I, Isoleucine; P, Proline; F, Phenylalanine; Y, Tyrosine; W, Tryptophan; S, Serine; T, Threonine; C, Cystine; M, Methionine; N, Asparagine; Q, Glutamine; D, Aspartic acid; E, Glutamic acid; K, Lysine; R, Arginine; H, Histidine. **(B)** The spatial structures of HSA and BSA. (PDB ID of HSA: 1AO6; PDB ID of BSA: 3V03). The molecular weight of BSA is 66432.96 Da, as calculated using the Expasy ProtParam tool from http://www.expasy.org/tools/protparam.html.

HSA and BSA show a high similarity in the amino acid residues and the three-dimensional structure (**Figure S2**). Considering the cost of the experiments, the BSA was chosen in the present study.

There were two peaks in matrix-assisted laser desorption ionization time-of-flight (MALDI-TOF) mass spectrometry of BSA (**Figure S3**), the mass-to-charge ratio *m*/*z* of nearly 66600 and 33300 Da, which may be contribute to BSA^{+} and BSA^{2+}, so the molecular weight (MW) of BSA was nearly 66600 Da. The MW was close to the theoretical value 66432.96 Da (**Figure S2A**).

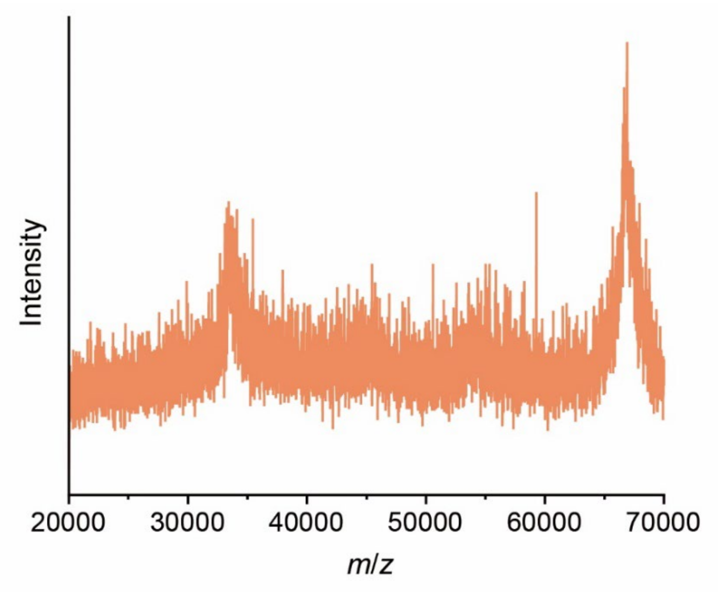

Figure S3. MALDI-TOF mass spectrum of BSA at positive mode. Sinapic acid was used as the MALDI matrix.

The impurities of iron, magnesium and zinc ions in BSA (**Table S2**) were very low (all below 0.4 μg/g), indicating the little interference on the following corrosion experiments.

Table S2. The impurity contents of metal ions in BSA

Impurities	Content (μg/g)
Iron	0.011
Magnesium	0.355
Zinc	0.025

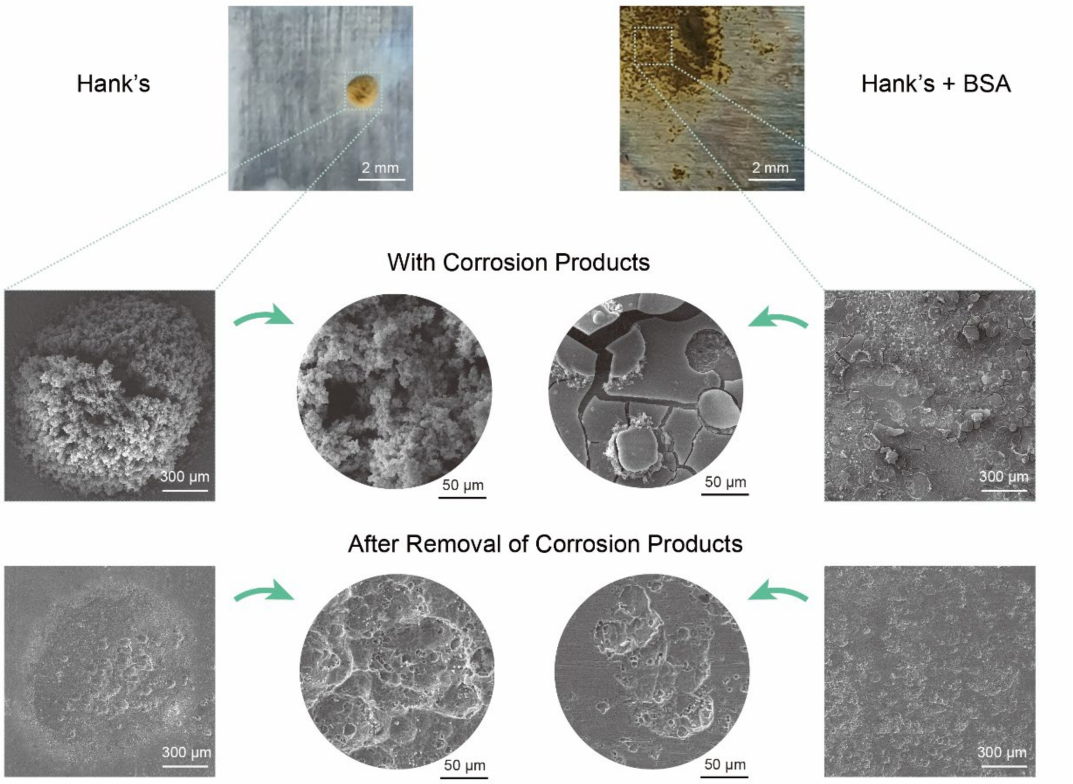

Figure S4. The optical photographs and the scanning electron microscopy (SEM) images of the iron sheets (10 mm × 10 mm × 0.2 mm) after being immersed in Hank's solution with and without 40.0 g/L BSA for 24 h. The iron sheets were ultrasonic cleaned in tartaric acid (4 wt.%) for 20 s to remove corrosion products.

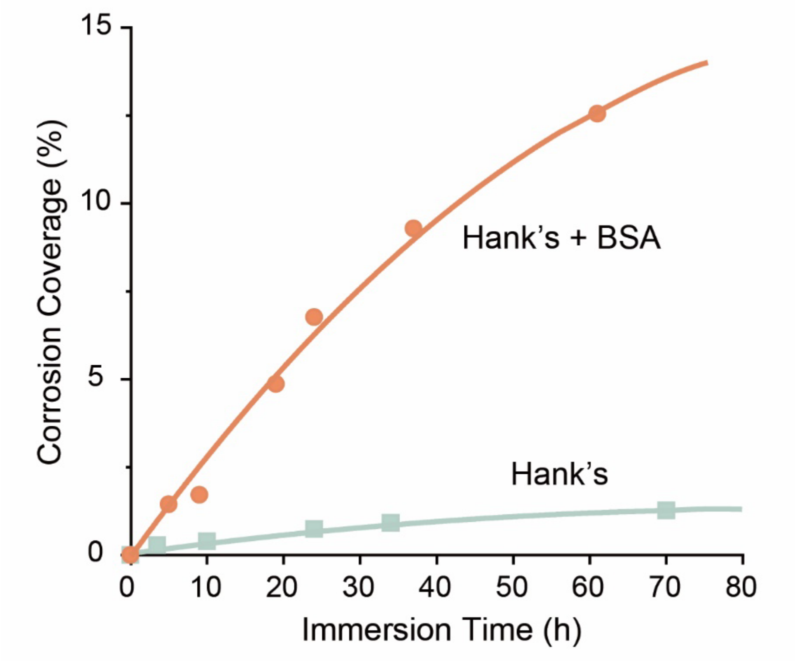

Figure S5. The corrosion coverage of iron sheets after being immersed in Hank's solution with and without 40.0 g/L BSA for the indicated time. The corrosion coverage was acquired by the SEM images after removal of corrosion products.

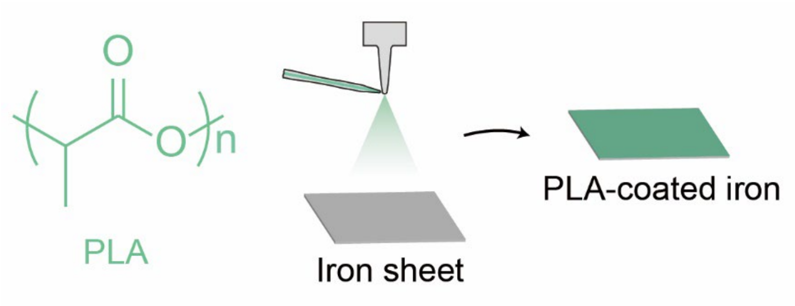

Figure S6. Illustration of ultrasonically spraying of a polylactide (PLA) solution to prepare the PLA-coated iron sheets.

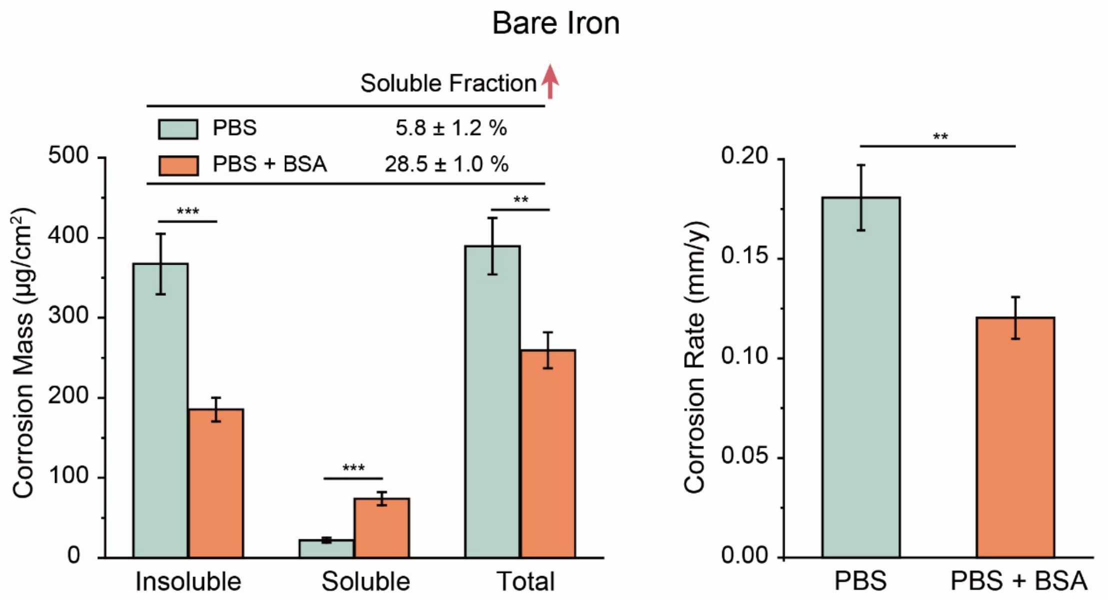

Figure S7. Corrosion rates and the mass of insoluble, soluble and total corrosion products of bare iron sheets after being immersed in phosphate buffer saline (PBS) solutions with and without 40.0 g/L BSA for 24 h. The corrosion rate was calculated based on the corrosion mass at 24 h and the metal density. The red arrow highlights the increase of the fraction of the soluble corrosion product of iron in the presence of BSA. ("**", $p < 0.01$; "***", $p < 0.001$; $n = 4$.)

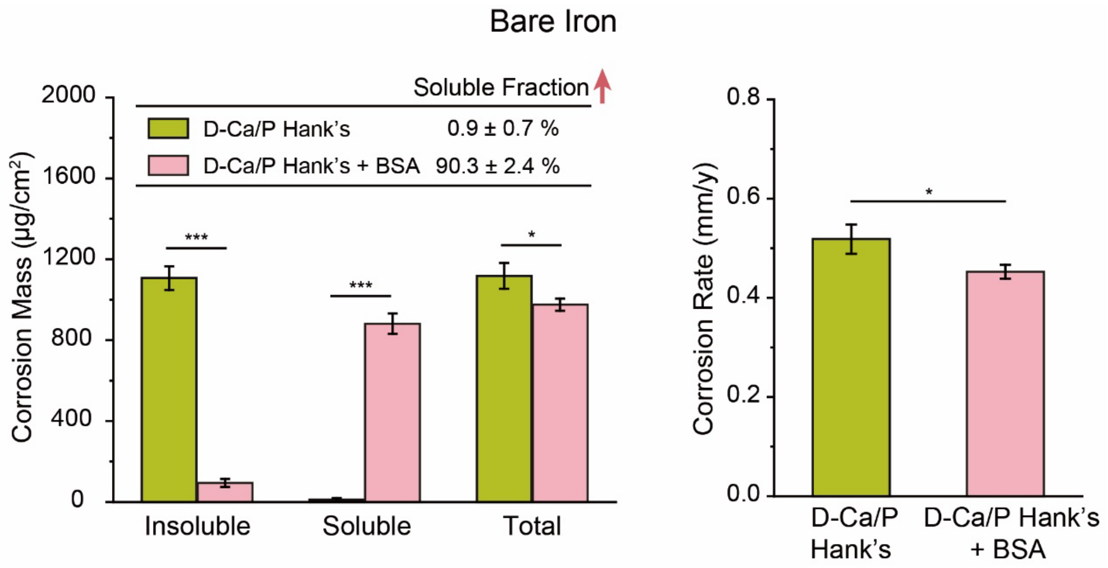

Figure S8. Corrosion rates and the mass of insoluble, soluble and total corrosion products of bare iron sheets after being immersed in D-Ca/P Hank's solution with and without 40.0 g/L BSA for 24 h. The corrosion rate was calculated from the corrosion mass at 24 h and the metal density. The red arrow indicates the increase of the soluble fraction. ("*", $p < 0.05$; "***", $p < 0.001$; $n = 4$.)

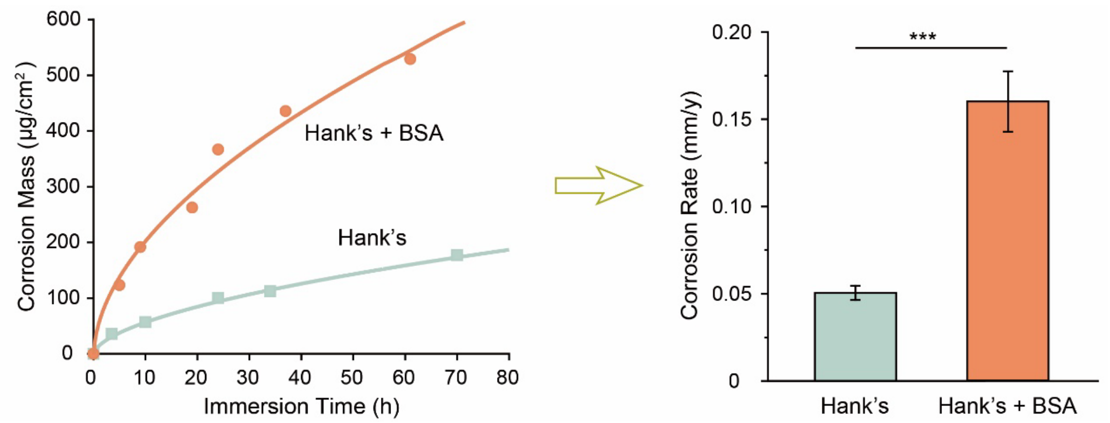

Figure S9. The corrosion mass as a function of time (left) and the corrosion rate (right) of iron sheets after being immersed in Hank's solution with and without BSA. The corrosion mass was quantified based the exposed area of iron sheets and the corrosion amount of iron measured by inductively coupled plasma atomic emission spectrometry (ICP-AES). The corrosion rate was calculated based on the corrosion mass at 24 h. The corrosion mass of iron sheets immersed in Hank's solution with BSA was greater than that without BSA, and the corrosion rate during the initial 24 h was increased nearly 3 times, indicating that albumin accelerated the corrosion of iron. ("***", $p < 0.001$; n = 4.)

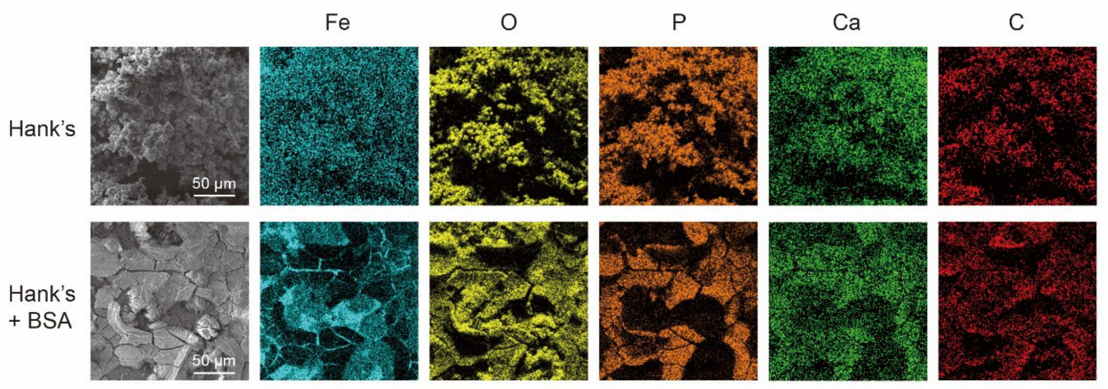

Figure S10. Scanning electron microscopy-energy dispersive spectrometer (SEM-EDS) images of the iron sheets after being immersed in Hank's solution with and without 40.0 g/L BSA for 24 h.

Table S3. The surface atomic composition of the iron sheets characterized by EDS after being immersed in Hank's solution with and without 40.0 g/L BSA for 24 h.

	Fe/%	O/%	Ca/%	P/%	C/%
Hank's	30.2	47.0	2.1	9.7	11.0
Hank's + BSA	39.8	42.8	0.9	3.7	12.8

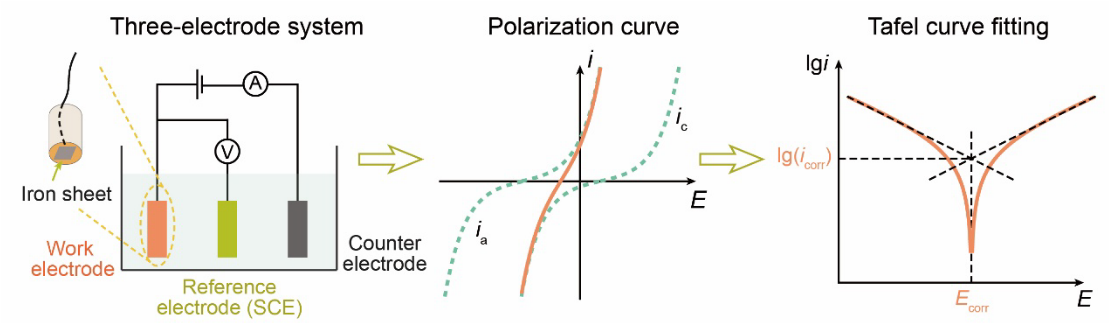

Figure S11. Schematic diagram of three-electrode system (left), potentiodynamic polarization curve (middle) and Tafel curve fitting (right) for the determination of corrosion current density (i_{corr}). The terms i_a and i_c represent the current densities of anodic and cathodic reactions, respectively.

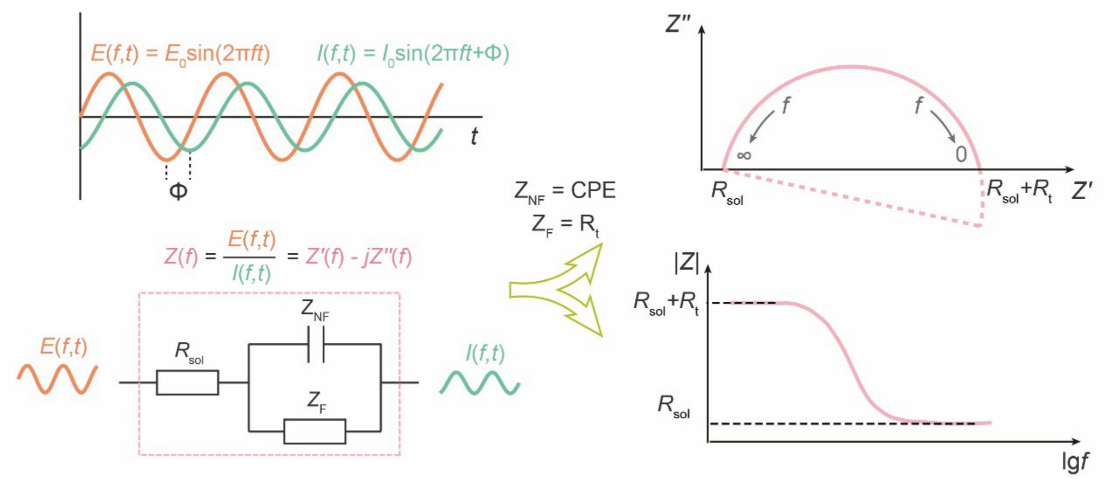

Figure S12. Schematic diagram of electrochemical impedance spectroscopy which is a powerful method to monitor the kinetics in the electrode process. A sinusoidal voltage $E(f,t) = E_0\sin(2\pi ft)$ was applied to the tested electrode system with frequency f. The perturbation amplitude of the voltage was small to get a linear response of the resultant current density, expressed as $I(f,t) = I_0\sin(2\pi ft + \Phi)$, where Φ represents the phase angle. The impedance Z of the system could be obtained by voltage and current. It is divided into two parts: real impedance Z' and imaginary impedance Z''. The impedance Z could be expressed by an equivalent circuit with Faraday impedance Z_F, non-Faraday impedance Z_{NF} and solution resistance R_{sol}. Z_F is commonly represented by a constant phase element (CPE) and Z_{NF} by a transfer resistance R_t of double electric layer. By varying the signal frequency f, the R_t could be obtained by the spectrum of complex impedance Z, such as Nyquist plots and Bode plots. The impedance as the frequency f approaches infinity is equal to R_t while the impedance as the frequency f approaches zero is equal to $R_t + R_{sol}$.

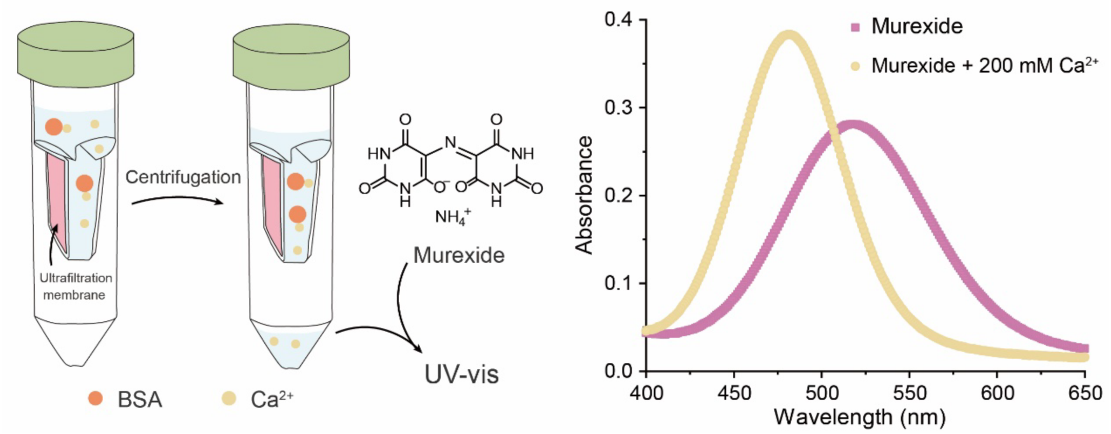

Figure S13. Determination of the free Ca^{2+} concentration in the calcium-containing solutions. (Left) Schematic diagram of the method to determine the free Ca^{2+} concentration using murexide and ultraviolet-visible (UV-vis) spectroscopy. The peak of murexide moved from 520 nm to 480 nm after bounded with Ca^{2+}. Murexide suffers from its downside that once bound to the albumin the quantification is inaccurate, so free Ca^{2+} was separated by a centrifugal filter to avoid the binding of albumin to murexide. (Right) UV-vis spectrum of free and bound murexide at 37°C. The concentration of murexide was 0.03 mM.

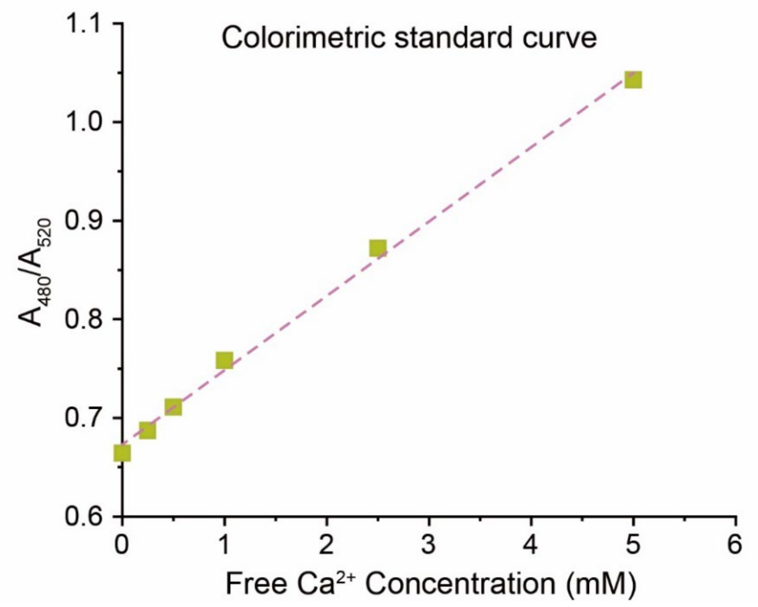

Figure S14. Standard curve for determination of free Ca^{2+} in the calcium-containing solutions by the method using murexide. The absorbance ratio of 480 nm to 520 nm showed a linear relationship between free Ca^{2+} concentration in a certain range

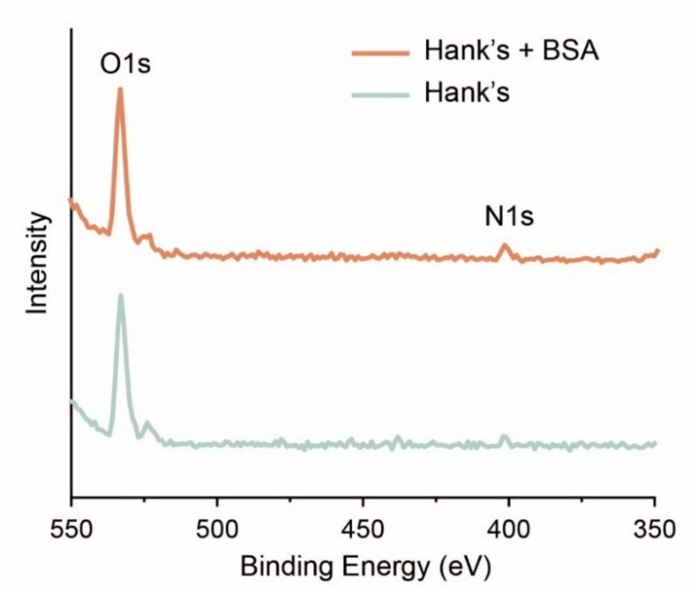

Figure S15. X-ray photoelectron spectra (XPS) of iron sheets after being immersed in Hank's solution with and without 40.0 g/L BSA for 24 h.

Table S4. The surface atomic composition of the iron sheets characterized by XPS after being immersed in Hank's solution with and without 40.0 g/L BSA for 24 h.

	N/%	C/%	O/%	Fe/%	Ca/%	P/%
Hank's	2.8	59.8	35.3	0.6	0.7	0.8
Hank's + BSA	5.7	47.8	38.9	3.9	1.1	2.6

We carried out atomic force microscopy (AFM) imaging to visualize the adsorbed albumin. From **Figure S16A**, there were many corrosion product particles on the iron surface, which made AFM imaging difficult to distinguish adsorbed albumin.

To avoid the effects of corrosion, the 316L stainless steels were applied. Compared with unimmersed stainless steel, the surface immersed in Hank's solution was full of larger particles of about 10 nm, which may be the deposited Ca/P salts (**Figure S16B**). On the surface immersed in Hank's solution with BSA, the particles were larger, which may be a mixture of the deposited Ca/P salts and adsorbed albumin aggregates. The above results were obtained in air, where the drying process may lead to protein denaturation, making the *in situ* observation in the fluid more necessary.

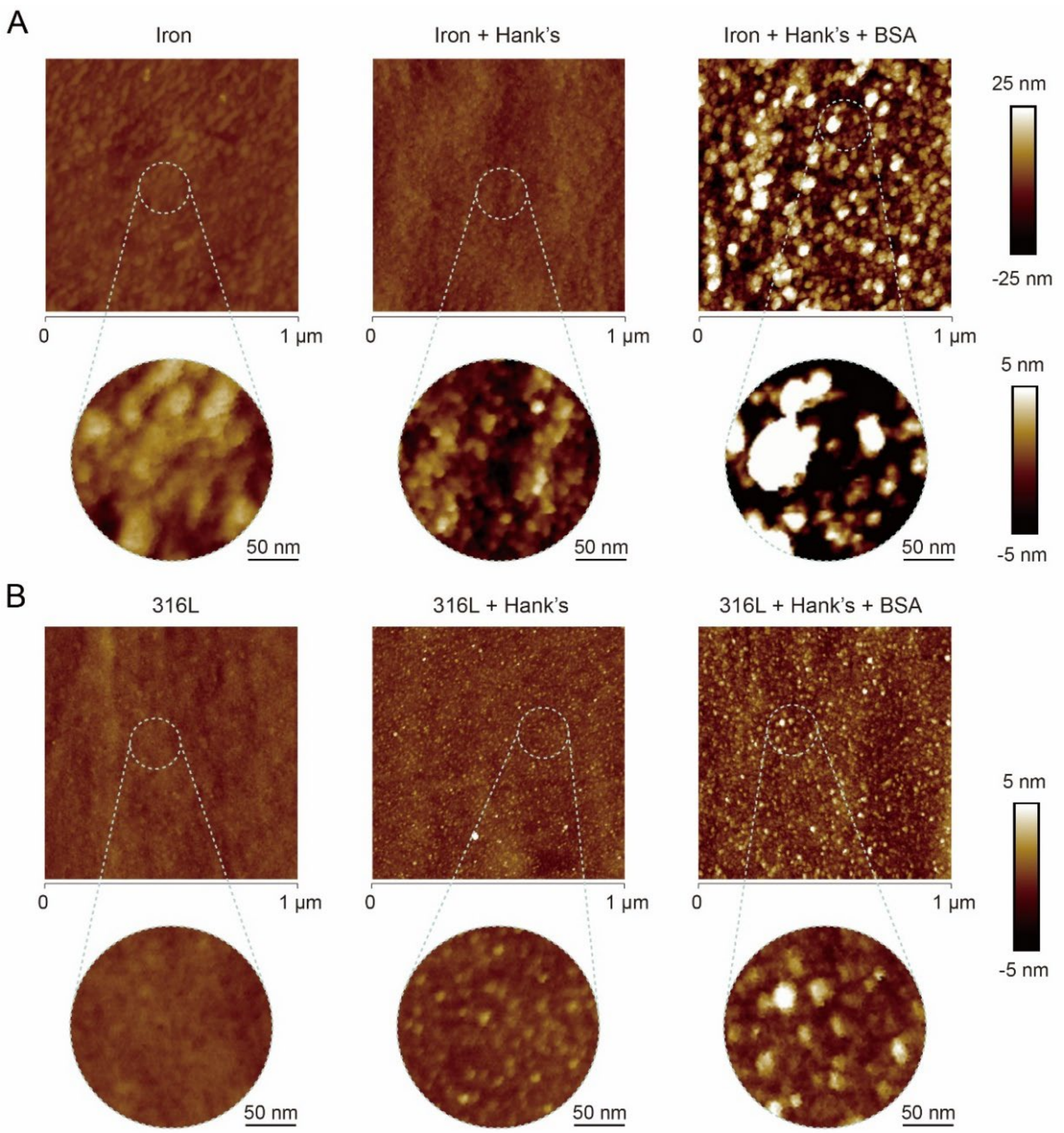

Figure S16. **Characterization of the morphology of adsorbed albumin in air. (A)** AFM images of the unimmersed iron sheet and the iron sheets after being immersed in Hank's solution with and without 40.0 g/L BSA for 10 min. **(B)** AFM images of the unimmersed 316L stainless steel sheet and the 316L stainless steel sheets after being immersed in Hank's solution with and without 40.0 g/L BSA for 24 h.

Eventually, our AFM imaging was made mainly under an aqueous medium. From **Figure S17**, the surfaces of iron sheets were full of large particles of corrosion products when being immersed from 10 to 15 min whether with albumin or not, similar to the result of **Figure S16A**.

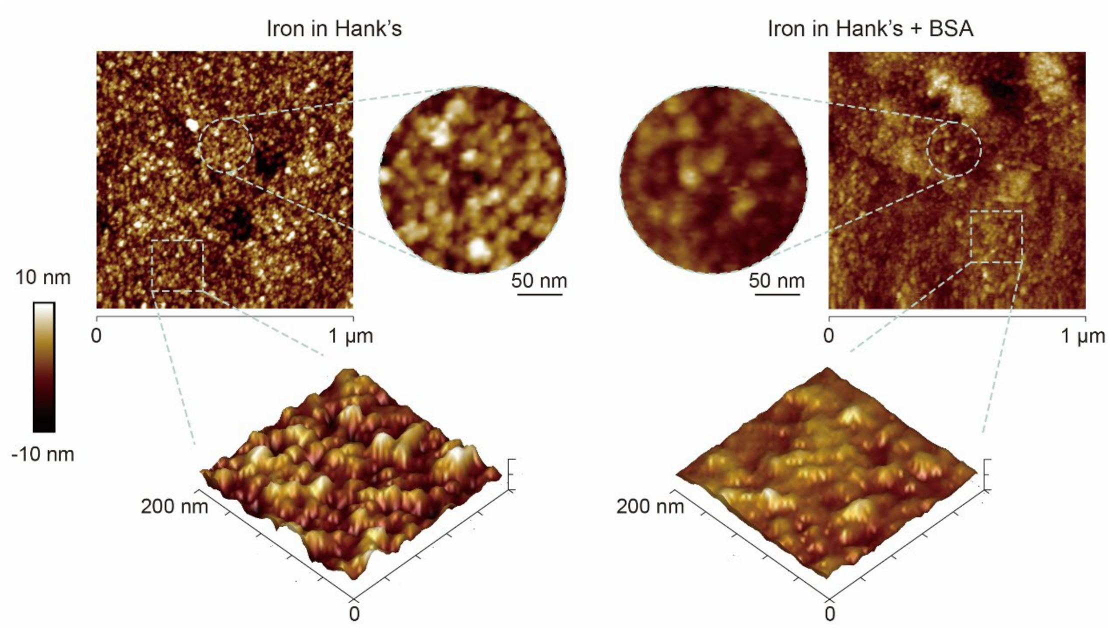

Figure S17. *In situ* AFM images of the iron sheets immersed in Hank's solution with and without 40.0 g/L BSA from 10 to 15 min. The *Z* range of 3D images of iron was from -10 nm to 10 nm and the *Z*-axis was magnified twice.

Mica was applied to get a flat base, and the D-Ca/P Hank's solution was applied to avoid interference from deposited Ca/P salts, as demonstrated by **Figure S18A**. When being immersed with BSA from 10 to 15 min, a protein membrane with some aggregates formed on the surface (**Figure S18B**). The membrane changed over time, showing both of the appearance and disappearance of the aggregates.

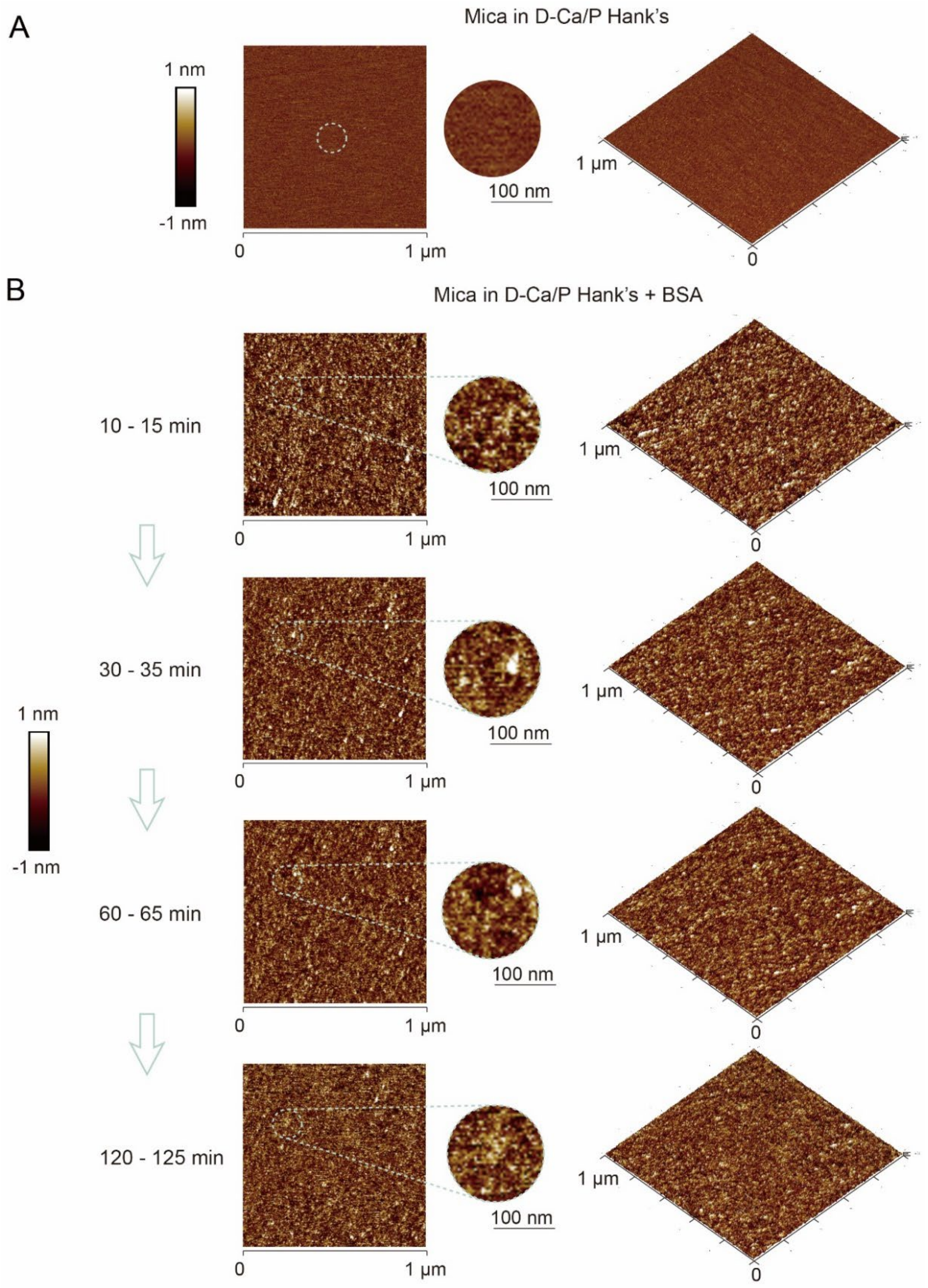

Figure S18. *In situ* characterization of the morphology of adsorbed layers of albumin in fluid. (A) AFM images of the mica immersed in D-Ca/P Hank's solution. **(B)** *In situ* AFM images of mica immersed in D-Ca/P Hank's with 40.0 g/L BSA from 10 to 125 min. The Z range of the 3D images was from -1 nm to 1 nm and the Z-axis was magnified 10 times.

In order to observe the morphology of single molecular albumin, mica was treated with Ni^{2+} and the protein solution was diluted. When immersed in BSA aqueous solution from 10 to 15 min, there were many nanoparticles on the surface of Ni-mica (**Figure S19**), representing the single molecular BSA.

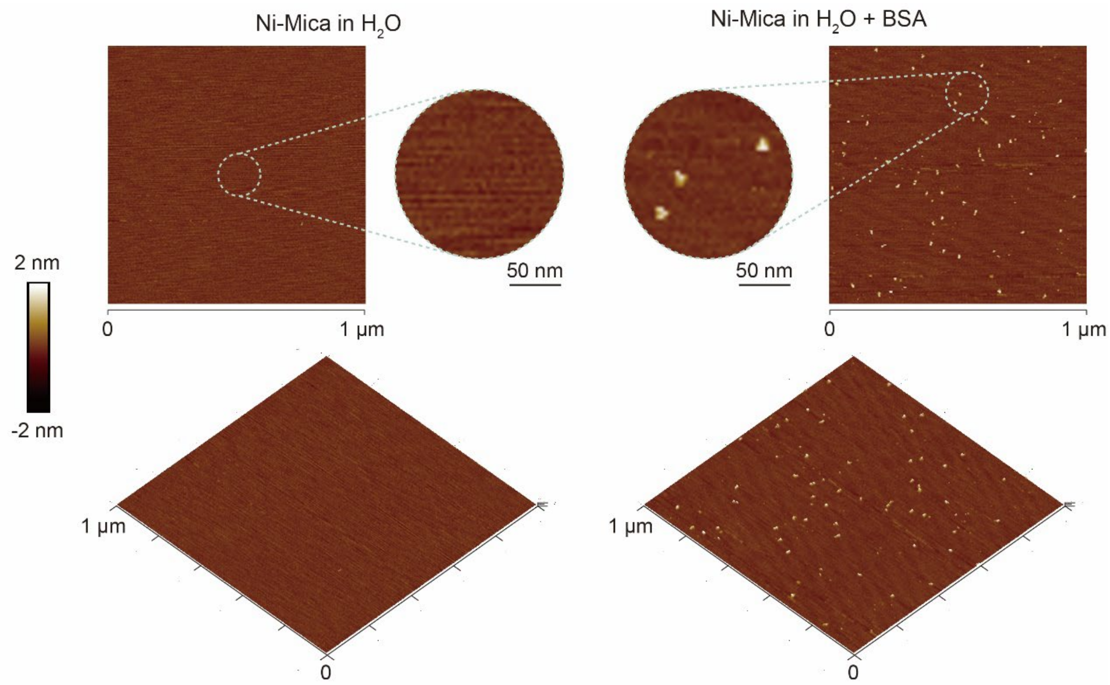

Figure S19. *In situ* characterization of the morphology of adsorbed single molecule albumin in fluid. (Left) AFM images of the Ni-mica immersed in H_2O. (Right) *In situ* AFM images of Ni-mica immersed in 4 mg/L BSA aqueous solution from 10 min to 15 min. The *Z* range of 3D images was from -2 nm to 2 nm and the *Z*-axis was magnified 4 times.

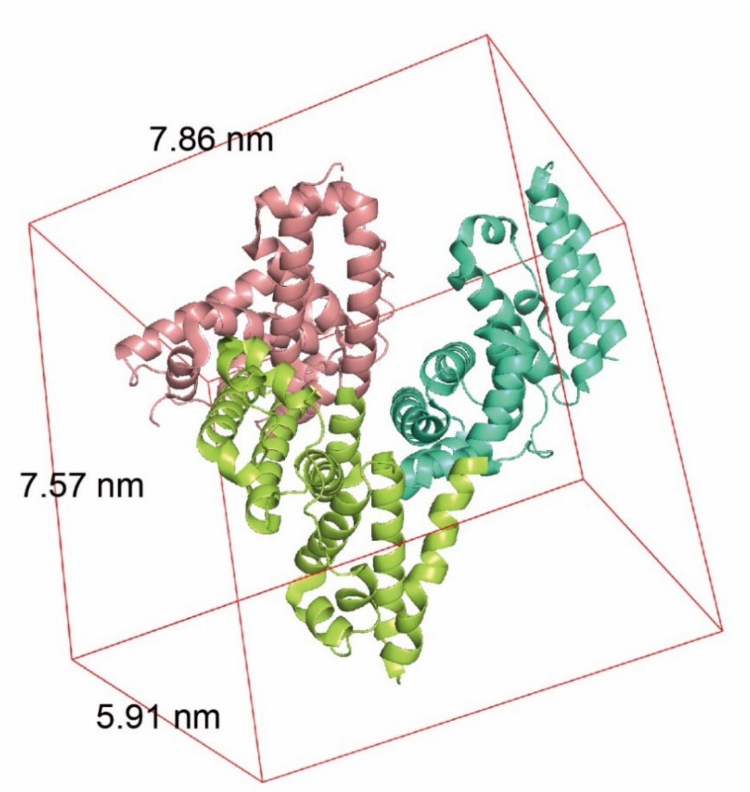

Figure S20. The size of a BSA molecule (PDB ID: 3V03).

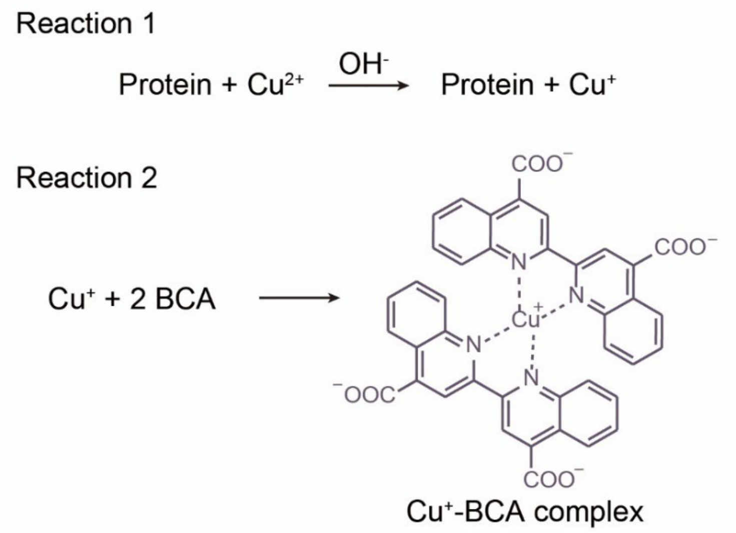

Figure S21. The principle of micro-bicinchoninic acid (BCA) assay to quantify the concentration of proteins. The Cu^{2+} is reduced to Cu^{+} by protein under alkaline condition, then the Cu^{+} is chelated by BCA to form a purple complex, which is proportional to the amount of protein.

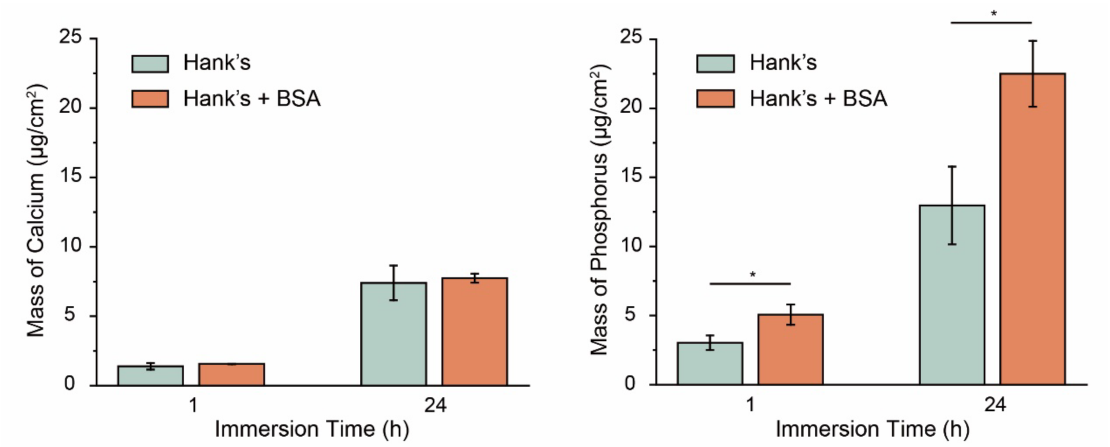

Figure S22. The mass of calcium (left) and phosphorus (right) on the surface of iron sheets immersed in Hank's solution with and without 40.0 g/L BSA for 1 h and 24 h. The calcium and phosphorus on the surface were dissolved in nitric acid (5 wt.%), and the mass were quantified by ICP-AES. ("*", $p < 0.05$; $n = 3$.)

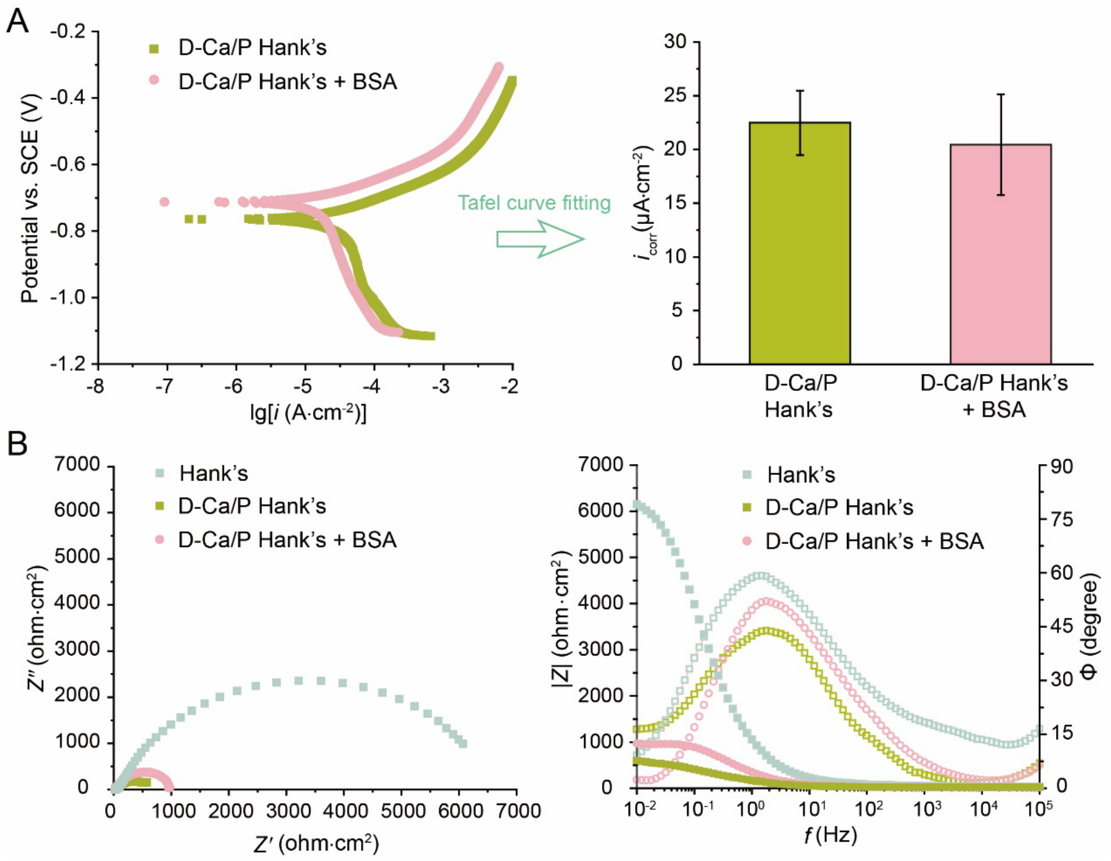

Figure S23. Electrochemical tests of iron electrodes immersed in D-Ca/P Hank's solution with and without BSA. (A) Tafel curves (left) and corrosion current densities (right) of iron electrodes after being immersed for 24 h. **(B)** Nyquist plots (left) and Bode plots (right) of the iron electrodes after being immersed in Hank's solution and D-Ca/P Hank's solution with and without 40.0 g/L BSA for 24 h.
